# Supplementary material for: Influenza Virus Mounts a Two-Pronged Attack on Host RNA Polymerase II Transcription
Source: Cell Rep. 2018 May 15;23(7):2119–2129.e3. doi: 10.1016/j.celrep.2018.04.047 (PMC5972227; doi:10.1016/j.celrep.2018.04.047)
Supplement: Document S1. Figures S1–S4 and Table S2 [file mmc1.pdf]

**Cell Reports, Volume *23***

## Supplemental Information

# Influenza Virus Mounts a Two-Pronged Attack on Host RNA Polymerase II Transcription

**David L.V. Bauer, Michael Tellier, Mónica Martínez-Alonso, Takayuki Nojima, Nick J. Proudfoot, Shona Murphy, and Ervin Fodor**

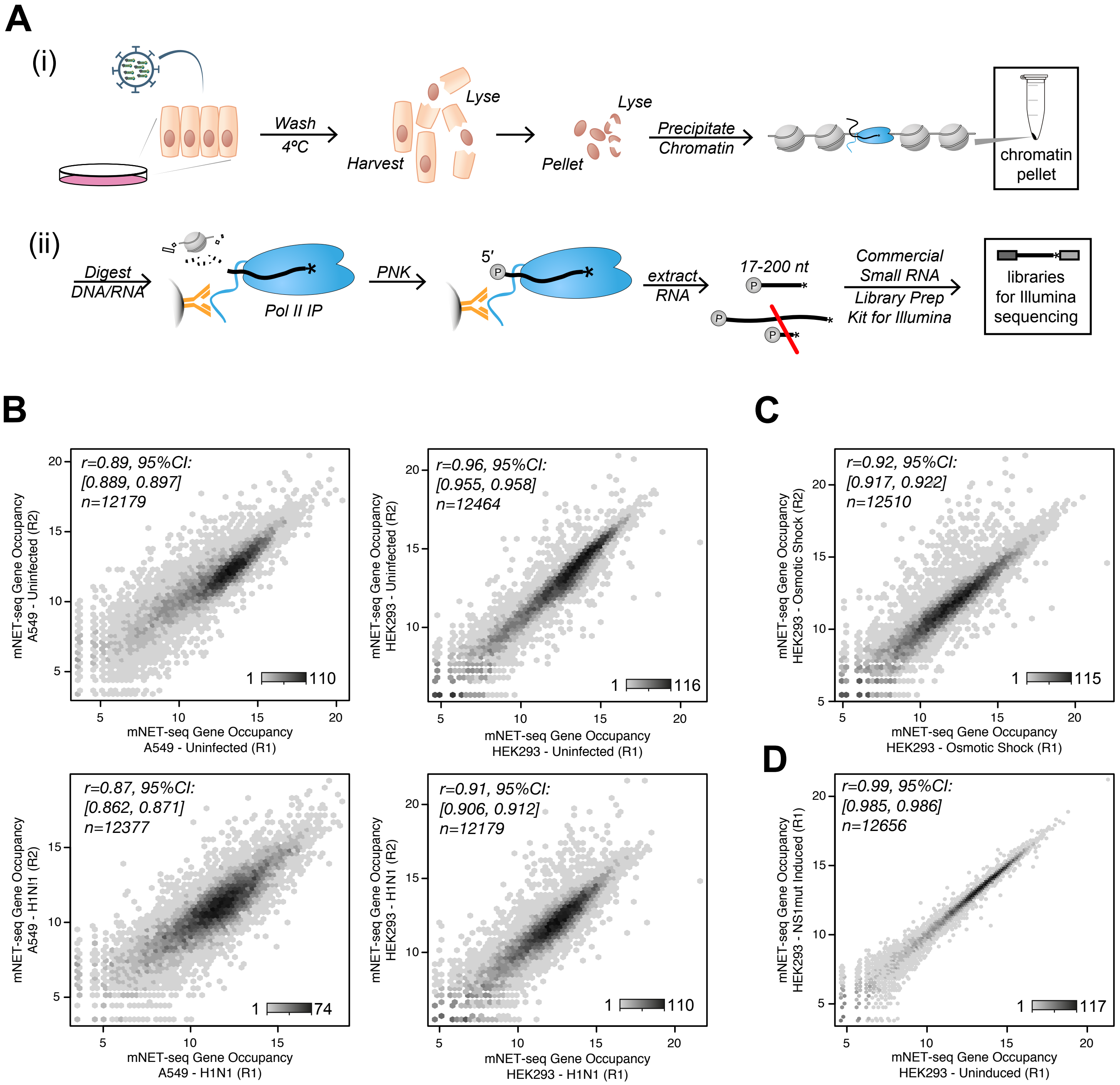


**Figure S1. Mammalian Nascent Elongating Transcript - Sequencing (mNET-seq) Generates Highly Reproducible Profiles of Pol II Occupancy Across the Genome. Related to Figure 1**

(A) Outline of mNET-seq (i) sample processing and (ii) library preparation, based on the method described in (Nojima et al., 2015) and the protocol described in detail in (Nojima et al., 2016). See STAR Methods for further details.

(B) Comparison of mNET-seq results (gene body Pol II occupancy) between biological replicates (R1 and R2) in A549 (left) and HEK293 (right) cells either uninfected (top) or H1N1-infected (bottom).

(C) Comparison of mNET-seq results (gene body Pol II occupancy) between biological replicates of osmotic shock treatment in HEK293 cells.

(D) Comparison of mNET-seq results (gene body Pol II occupancy) between uninduced HEK293 cells and cells in which expression of a CPSF30 binding mutant of the influenza virus NS1 protein (NS1mut) was induced.

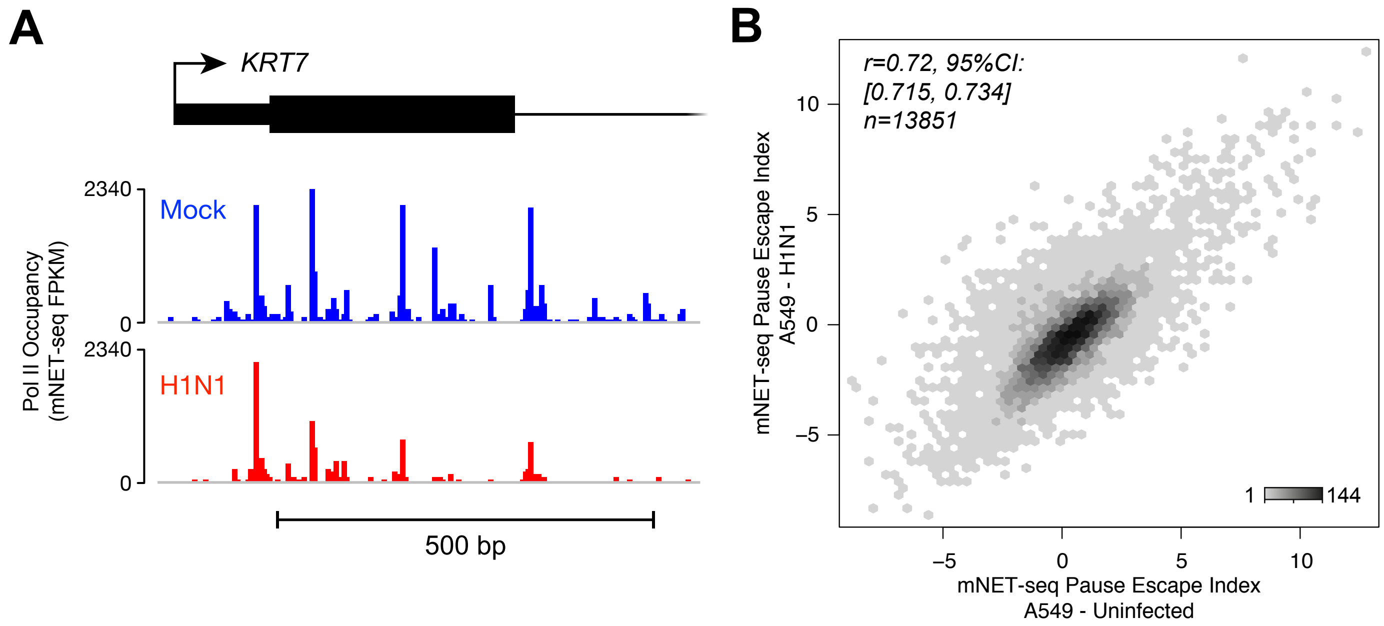


**Figure S2. Pol II Occupancy Tails Off Downstream of Transcription Start Sites During Influenza Infection. Related to Figure 2.**

(A) mNET-seq profiles of Pol II occupancy at the start of the protein-coding gene *KRT7*. Influenza virus infection (H1N1) causes a 'tailing-off' of Pol II occupancy in the body of the gene.

(B) The Pause Escape Index (ratio between the amount of promoter-proximally paused Pol II in the TSS region and amount of Pol II in the gene body, see STAR Methods) was calculated for each gene, and compared between uninfected and H1N1-infected samples. The linear correlation (Pearson's r=0.72, 95% confidence interval = [0.715, 0.734]) reflects the 'tailing-off' of Pol II and also suggests that FluPol does not preferentially target genes with different amounts of promoter-proximally-paused Pol II, which would generate a non-linear correlation.

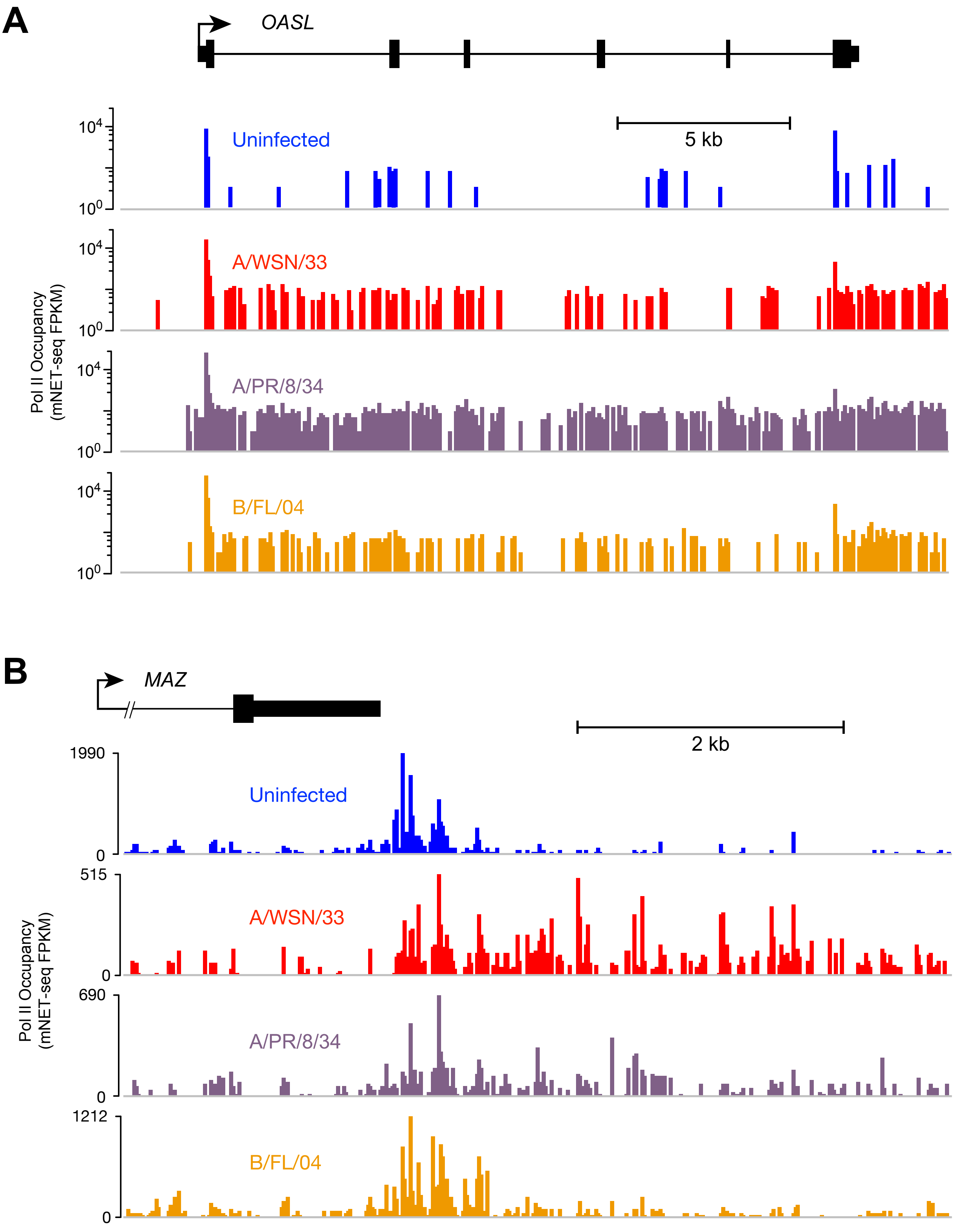


**Figure S3. Transcriptional Response to Influenza Virus Infection. Related to Figure 5.**
(A) mNET-seq profiles of Pol II occupancy along the antiviral gene *OASL* , in uninfected and infected (A/WSN/33, A/PR/8/34, and B/Florida/04) cells. Pol II occupancy along *OASL* is increased during viral infection, suggesting that cells respond to viral infection. Note that the y-axis is presented on a logarithmic scale.
(B) mNET-seq profiles of Pol II occupancy along the protein-coding gene *MAZ* show the same pattern of termination defect as on *KRT7* (Figure 5). The unexpressed downstream *PRRT2* gene is not shown in the annotation.

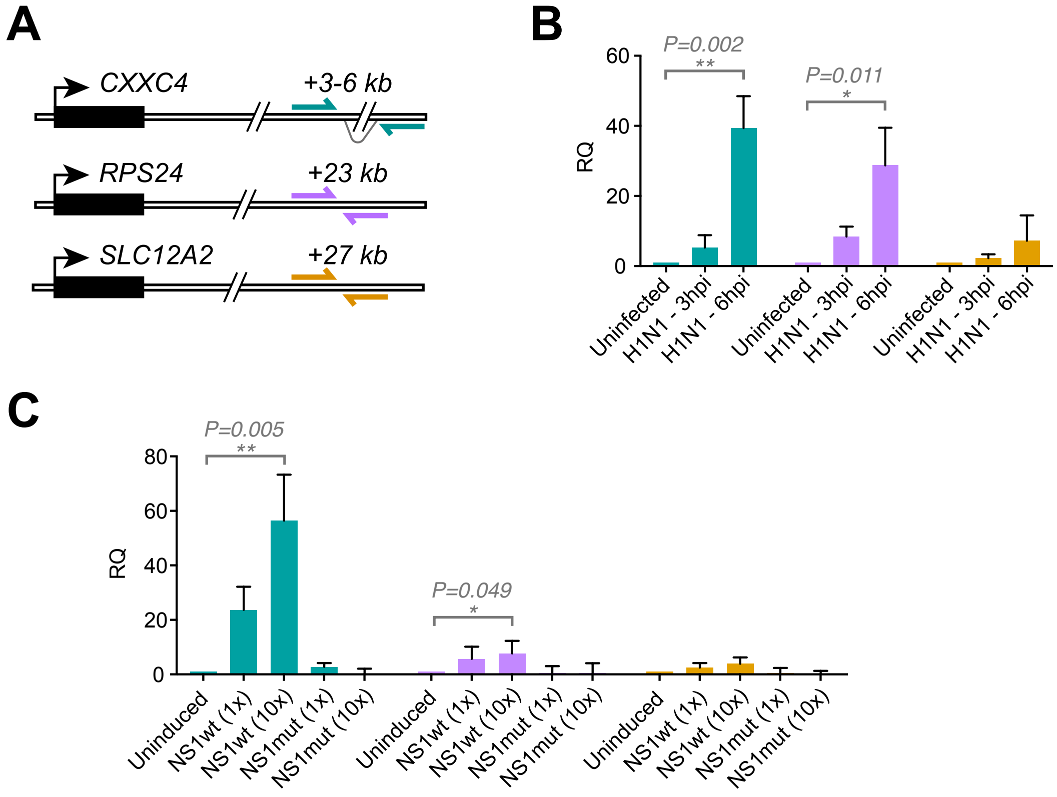


**Figure S4. Downstream-of-Gene transcripts (DoGs) Are Produced During Influenza Virus Infection and Viral NS1 Protein Expression. Related to Figure 6.**
(A) RT-qPCR scheme for the detection of DoGs (see STAR Methods) downstream of *CXXC4*, *RPS24*, and *SLC12A2*. The grey connecting arc downstream of *CXXC4* represents a reported splice junction (Vilborg et al., 2015).
(B) Quantitation of DoGs present during influenza virus infection in A549 cells at 3 and 6 hours post infection (hpi). Error bars represent standard deviation from three biological replicates.
(C) Quantitation of DoGs present following induction of NS1wt or NS1mut protein expression in HEK293 cells. (1x) indicates 1 µg/ml tetracycline, (10x) indicates 10 µg/ml tetracycline used to induce protein expression. Error bars represent standard deviation from three biological replicates.

| Name | Sequence (5′ to 3′) | Reference |
|---|---|---|
| doCXXC4F | CAGTGAAGCTCTTCGATTTCAA | (Vilborg et al., 2015) |
| doCXXC4R | CCCCCTATATACCGAAGTGGA | (Vilborg et al., 2015) |
| SLC12A2_tdF | GAAACTGCTGTTGGAAGCGG | |
| SLC12A2_tdR | TGGCCTTGGATTAGCATCCG | |
| RPS24_td23_F | CTTGGATGCCATGTTGCCAG | |
| RPS24_td23_R | CAGACCAAAAGCGGAGCCTA | |
| 18s_F | ACCCGTTGAACCCCATTCGTGA | |
| 18s_R | GCCTCACTAAACCATCCAATCGG | |

**Table S2. Primer oligonucleotide sequences used for RT-qPCR detection of DoG transcripts. Related to STAR Methods.**
