# Supplementary material for: Influenza Virus Mounts a Two-Pronged Attack on Host RNA Polymerase II Transcription
Source: Cell Rep. 2018 May 15;23(7):2119–2129.e3. doi: 10.1016/j.celrep.2018.04.047 (PMC5972227; doi:10.1016/j.celrep.2018.04.047)
Supplement: Document S2. Article plus Supplemental Information [file mmc3.pdf]

Cell Reports

Article

Influenza Virus Mounts a Two-Pronged Attack on Host RNA Polymerase II Transcription

Graphical Abstract

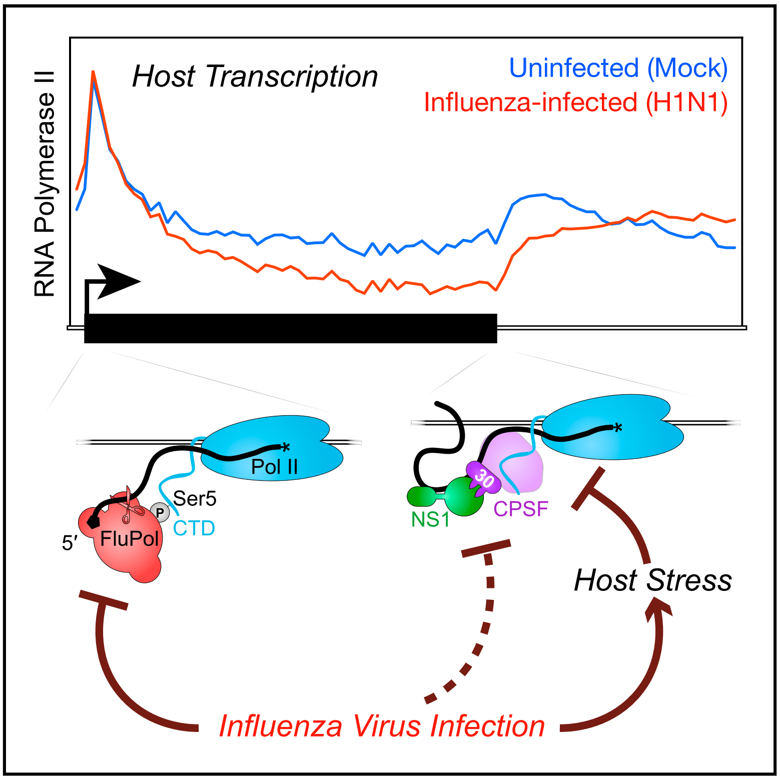

Highlights

- Influenza virus infection dysregulates host transcription
- Viral infection depletes Pol II from gene bodies downstream of the TSS
- Virus-induced stress leads to a catastrophic failure of Pol II termination
- Defective termination does not require viral NS1: host CPSF30 interaction

Authors

David L.V. Bauer, Michael Tellier, Mónica Martínez-Alonso, Takayuki Nojima, Nick J. Proudfoot, Shona Murphy, Ervin Fodor

Correspondence

david.bauer@path.ox.ac.uk (D.L.V.B.), ervin.fodor@path.ox.ac.uk (E.F.)

In Brief

Bauer et al. investigate the effects of influenza virus infection on host RNA polymerase II (Pol II) transcription genome-wide. They find that infection leads to dysregulation at both the starts and ends of genes. Their work provides insight into both virus-host interactions and fundamental mechanisms of mammalian transcription.

Bauer et al., 2018, Cell Reports *23*, 2119–2129
May 15, 2018 © 2018 The Author(s).
https://doi.org/10.1016/j.celrep.2018.04.047

CellPress

Cell Reports
Article

OPEN ACCESS
CellPress

Influenza Virus Mounts a Two-Pronged Attack on Host RNA Polymerase II Transcription

David L.V. Bauer,[1,*] Michael Tellier,[1] Mónica Martínez-Alonso,[1] Takayuki Nojima,[1] Nick J. Proudfoot,[1] Shona Murphy,[1] and Ervin Fodor[1,2,*]
[1]Sir William Dunn School of Pathology, University of Oxford, South Parks Road, Oxford OX1 3RE, UK
[2]Lead Contact
*Correspondence: david.bauer@path.ox.ac.uk (D.L.V.B.), ervin.fodor@path.ox.ac.uk (E.F.)
https://doi.org/10.1016/j.celrep.2018.04.047

SUMMARY

Influenza virus intimately associates with host RNA polymerase II (Pol II) and mRNA processing machinery. Here, we use mammalian native elongating transcript sequencing (mNET-seq) to examine Pol II behavior during viral infection. We show that influenza virus executes a two-pronged attack on host transcription. First, viral infection causes decreased Pol II gene occupancy downstream of transcription start sites. Second, virus-induced cellular stress leads to a catastrophic failure of Pol II termination at poly(A) sites, with transcription often continuing for tens of kilobases. Defective Pol II termination occurs independently of the ability of the viral NS1 protein to interfere with host mRNA processing. Instead, this termination defect is a common effect of diverse cellular stresses and underlies the production of previously reported downstream-of-gene transcripts (DoGs). Our work has implications for understanding not only host-virus interactions but also fundamental aspects of mammalian transcription.

INTRODUCTION

Influenza virus remains a challenge for global health and is responsible for both seasonal and pandemic outbreaks (Taubenberger and Kash, 2010). It has a negative-sense single-stranded segmented RNA genome and encodes its own RNA-dependent RNA polymerase (FluPol) that is responsible for carrying out both replication and transcription of the viral RNA genome in the nucleus of host cells (te Velthuis and Fodor, 2016). Both replication and transcription are carried out by FluPol in the context of viral ribonucleoprotein (vRNP) complexes (Eisfeld et al., 2015). For transcription, the virus is dependent on the host RNA polymerase II (Pol II) to supply 5′ capped transcripts, which are bound and cleaved by the cap-binding and endonuclease domains of FluPol. The resultant 5′ capped RNA fragments are then used as primers for viral mRNA synthesis (Krug et al., 1979). Accordingly, FluPol maintains a coordinated, intimate association with host Pol II.

The C-terminal domain of the large subunit of host Pol II (CTD) contains multiple repeats of the amino acid heptad: Tyr1-Ser2-Pro3-Thr4-Ser5-Pro6-Ser7, which are post-translationally modified in order to regulate and coordinate the transcription cycle and RNA processing and act as an assembly platform for various accessory enzymes (Zaborowska et al., 2016a). FluPol also binds to the Pol II CTD phosphorylated on Ser5 (Engelhardt et al., 2005; Lukarska et al., 2017; Martínez-Alonso et al., 2016). Phosphorylation of the Ser5 CTD residue (Ser5P) is a key marker for initiating Pol II and is specifically bound by the capping enzyme (Ho and Shuman, 1999), making the specificity of FluPol for the Ser5P form an efficient mechanism to target the pool of Pol II molecules associated with nascent, capped RNA substrates.

Viruses exploit a variety of mechanisms to prevent the host from responding to viral infection and often block transcription and translation of host genes (Herbert and Nag, 2016). Although there appears to be no preferential translation of influenza mRNA (Bercovich-Kinori et al., 2016), influenza virus infection interferes with transcription and mRNA processing to efficiently achieve a “takeover” of the mRNA pool. Although influenza virus cannot block transcription initiation and still obtain 5′ capped mRNA, infection inhibits Pol II elongation on β-actin and DHFR genes early in infection (Chan et al., 2006) and induces cellular Pol II degradation later in infection (Rodriguez et al., 2007; Vreede and Fodor, 2010; Vreede et al., 2010). A further opportunity for interference with host transcription lies in mRNA 3′ end processing, as influenza virus does not depend on the host cleavage and polyadenylation (CPA) machinery: FluPol synthesizes the poly(A) tail of viral mRNAs by repeated slippage on a poly(U) tract near the 5′ end of the viral RNA template (Poon et al., 1999). The nonstructural protein 1 (NS1) of some influenza virus strains interacts with human cleavage-polyadenylation specificity factor 30 (CPSF30) to restrict CPA of nascent host transcripts (Das et al., 2008; Davidson et al., 2014; Nemeroff et al., 1998), though this interaction is not conserved in other strains, including the well-studied A/Puerto Rico/8/34 influenza virus (Wang et al., 2017) and the 2009 pandemic H1N1 influenza A virus (Hale et al., 2010).

Although the studies above indicate that influenza virus infection can interfere with the Pol II transcription cycle at multiple stages, the global consequences for Pol II transcription remain unknown. Inhibition of transcriptional elongation and CPA suggest an effect on termination of Pol II, which is a highly coordinated and regulated process (Buratowski, 2005; Proudfoot, 2016; Richard and Manley, 2009). Indeed, recent genome-wide studies have shown that small interfering RNA (siRNA)-mediated depletion of CPSF73 or CstF64 results both in failure to process the 3′ ends of transcripts and in failure of Pol II to pause downstream of poly(A) sites, with Pol II continuing to transcribe past

© 2018 The Author(s).
This is an open access article under the CC BY license (http://creativecommons.org/licenses/by/4.0/).

normal transcription termination sites (Nojima et al., 2015). Infection with herpes simplex virus 1 (HSV-1), a double-stranded DNA virus, also induces a failure of Pol II to terminate, though the mechanism of action is unknown (Rutkowski et al., 2015). Surprisingly, multiple cellular stressors appear to generate defects in RNA 3′ end formation, with osmotic shock, heat shock, and oxidative stress all inducing the production of polyadenylated "downstream-of-gene" transcripts (DoGs) that extend for kilobases beyond normal poly(A) sites (Vilborg and Steitz, 2017; Vilborg et al., 2015, 2017). Although the purpose of this response and the function of these transcripts is unknown, it is suspected that defects in Pol II termination underlie their production (Mahat et al., 2016).

Given the intimate association between influenza virus and host transcription, we have explored the dynamics of Pol II during influenza virus infection using mammalian native elongating transcript sequencing (mNET-seq). We show that host transcription is drastically altered as a result of influenza virus infection and find that influenza virus mounts a two-pronged attack on host transcription. First, infection leads to decreased Pol II occupancy downstream of transcription start sites (TSSs), and second, interference with 3′ end processing leads to marked defects in termination of Pol II transcription at the end of genes.

RESULTS

Influenza Virus Infection Dramatically Alters the Transcription Dynamics of Host Pol II

To examine the effect of influenza virus infection on host Pol II transcription in human lung epithelial cells, we infected A549 cells at a high MOI (MOI = 5) with an H1N1 influenza virus (A/WSN/33) and carried out mammalian native elongating transcript sequencing (mNET-seq) to provide a single-nucleotide resolution map of Pol II activity genome-wide (Figures 1A and Figure S1). Examination of both mNET-seq meta-profiles of >13,000 protein-coding genes (Figure 1B) and profiles of individual genes (Figures 1C–1E) reveals two distinct effects. First, Pol II occupancy is markedly decreased in gene bodies relative to the TSS. Second, Pol II that has reached the gene end fails to terminate downstream of the poly(A) site and instead continues transcribing, often for tens of kilobases. We have systematically investigated these two effects in order to better understand the underlying molecular mechanisms with respect to both viral function and host cell transcription.

Influenza Virus Infection Causes Depletion of Pol II in Gene Bodies

We have examined the apparent depletion of Pol II downstream of TSSs in more detail to determine whether the effect is limited to particular genes and to understand the mechanism underlying the defect. We computed the Pol II occupancy (Nojima et al., 2015) observed by mNET-seq around the TSS of each gene and found that the mNET-seq Pol II signal is significantly depleted downstream of the TSS (Figure 2A). We hypothesized that this effect might arise as the result of FluPol association with Pol II, which specifically targets the Ser5P form of the Pol II CTD (Martínez-Alonso et al., 2016). We therefore carried out mNET-seq specifically for the Ser5P form of Pol II during influenza virus infection and examined the ratio of Ser5P Pol II to total Pol II along gene bodies (Figure 2B). Both infected and uninfected cells have an identical peak of Ser5P Pol II immediately downstream of the TSS, but influenza virus infection results in depletion of the Ser5P form of Pol II from chromatin further downstream, with Pol II occupancy tailing off into gene bodies, rather than occurring at a particular site (Figures S2A and S2B). We also found a more than 1,000-fold enrichment of influenza virus RNA co-immunoprecipitating with Ser5P Pol II relative to total Pol II in mNET-seq analysis (Figure 2C), mostly from the FluPol-bound termini of the vRNA that associated with Pol II and were protected from nuclease treatment during mNET-seq library preparation. This result confirms that FluPol colocalizes with Ser5P Pol II on chromatin in the context of vRNP complexes.

Widespread Failure of Pol II Termination in Influenza Virus-Infected Cells Is Linked to Canonical 3′ End Processing

We next turned our attention to the marked defect in host transcription termination during influenza virus infection. Pol II occupancy normally increases immediately downstream of the poly(A) sites of protein-coding genes as a result of Pol II pausing before termination (Glover-Cutter et al., 2008; Nojima et al., 2015). However, this is not the case during influenza virus infection (Figure 3A). We computed a Pol II read-through index for each gene to measure the degree to which Pol II fails to stop downstream poly(A) sites by calculating the ratio of mNET-seq reads in the 2 kb before and after the poly(A) site. We found that termination is significantly defective on protein-coding genes during influenza virus infection. On the other hand, termination is not defective on histone genes (Figures 3B and 3C), which are not processed by the canonical CPA complex (Kolev and Steitz, 2005). Together, these results suggest that the Pol II termination defect we observe arises from a result of interference with canonical mRNA 3′ end processing during influenza virus infection.

Expression of the Influenza Virus NS1 Protein Causes Widespread Failure of Pol II Termination

We considered whether the well-studied interaction between the influenza virus NS1 protein and host CPSF30 (Nemeroff et al., 1998) was responsible for the observed termination defect. We repeated our mNET-seq experiments with a HEK293 cell line expressing the viral NS1 protein under the control of a tetracycline-inducible promoter (Davidson et al., 2014) and compared the effects of NS1 protein alone with influenza virus infection (Figures 4A and 4B). We found that the expression of wild-type NS1 (but not that of a CPSF30-binding mutant) induced a similar Pol II termination defect as influenza virus infection, consistent with our results above. However, NS1 expression does not result in depletion of Pol II from gene bodies, suggesting that the termination defect at the ends of genes and the depletion of Pol II downstream of the TSS observed during influenza virus infection are distinct phenotypes. In line with these results, siRNA-mediated knockdown of CPSF30 also resulted in a significant Pol II termination defect (Figure 4C). Together, our data support the long-standing model of influenza interference with host transcription via an NS1-CPSF30 interaction (Nemeroff et al., 1998) and models of Pol II termination in which successful cleavage of

OPEN
ACCESS
CellPress

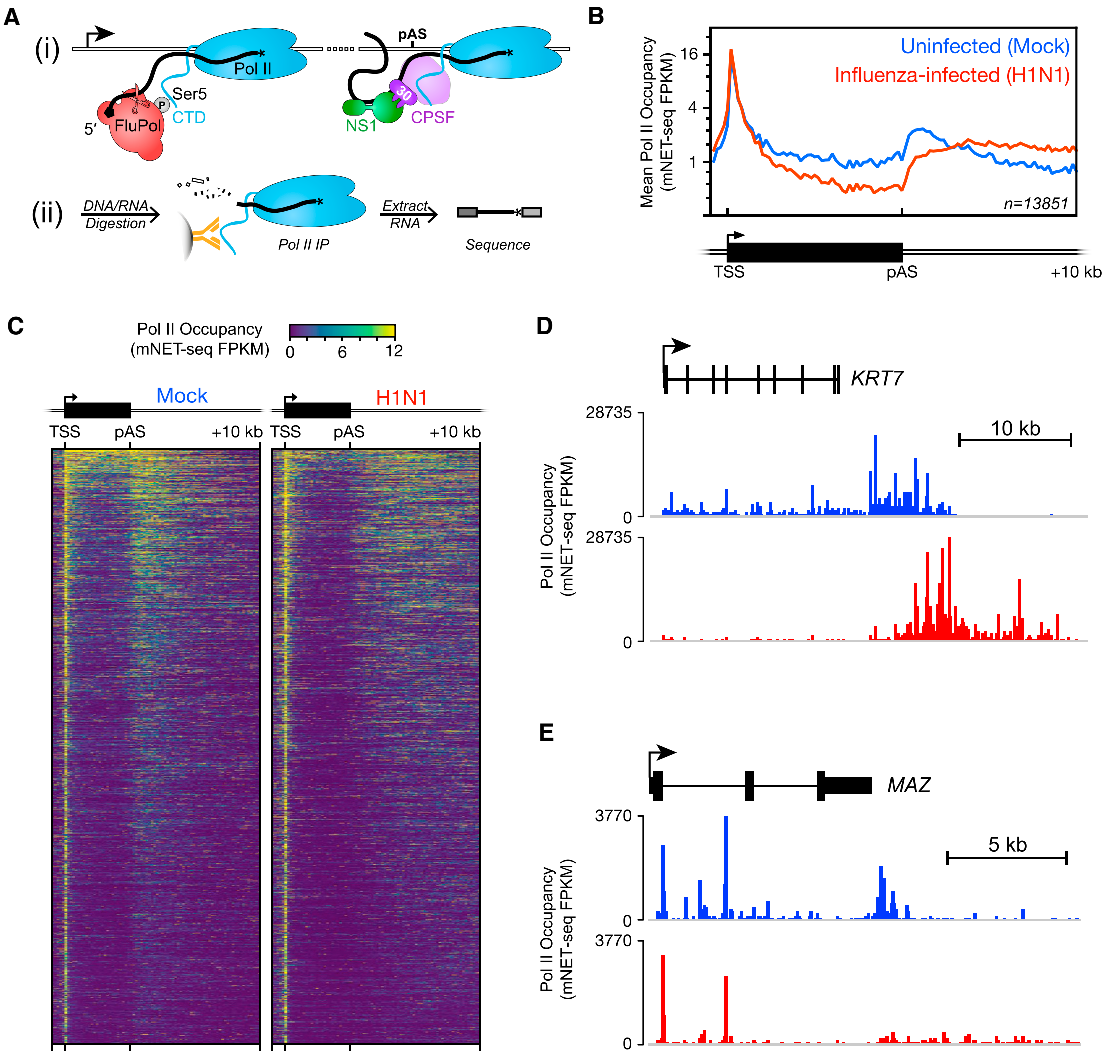

Figure 1. Influenza Virus Infection Dramatically Alters Transcription Dynamics of Host RNA Polymerase II

(A) (i) Influenza virus interferes with the host Pol II transcription complex, both at the start of genes where the viral polymerase (FluPol) binds to the Ser5P form of the Pol II CTD to carry out cap snatching, generating capped RNA primers for viral mRNA synthesis, and at the end of genes where the viral NS1 interferes with host 3′ end processing by inhibiting CPSF30. pAS, poly(A) site. (ii) Pol II behavior was profiled genome-wide using mNET-seq (see STAR Methods for details and Figure S1).

(B) Meta-profile of Pol II occupancy on all non-overlapping protein-coding genes (n = 13,851) as measured by mNET-seq shows that influenza virus infection simultaneously results in decreased Pol II occupancy downstream of the transcription start site (TSS), as well as a failure of Pol II to terminate downstream of the poly(A) site. FPKM, fragments per kilobase of transcript per million mapped reads.

(C) Heatmap of Pol II occupancy of non-overlapping protein-coding genes, with the TSS and poly(A) site of each gene aligned to each other, as illustrated at the top of the panel. Each horizontal line represents a single gene.

(D and E) mNET-seq profiles of Pol II occupancy along protein-coding genes *KRT7* (D) and *MAZ* (E). Downstream unexpressed genes (*KRT87P* and *PRRT2*) are not shown in the annotation.

In (B)–(E), data shown are from a single, representative biological replicate (see Figures S1B–S1D and Table S1 for comparison). See also Figure S1.

the transcript is a prerequisite (Proudfoot, 2016; West et al., 2004).

Influenza Virus Infection Causes a Widespread Pol II Termination Defect Independently of NS1-CPSF30 Interaction

Despite the considerable attention given to the canonical interaction between influenza virus NS1 and host CPSF30, the NS1 proteins of many influenza strains have mutations that prevent their binding to CPSF30 (Hale et al., 2010). In order to verify that the Pol II termination defect we observed during our influenza virus infections above was wholly due to the action of the viral NS1 protein, we carried out infections with an H1N1 influenza A virus strain (A/PR/8/34), closely related to A/WSN/33, expressing an NS1 protein that does not bind to CPSF30 (Das et al., 2008; Wang et al., 2017). We also tested an

OPEN
ACCESS
CellPress

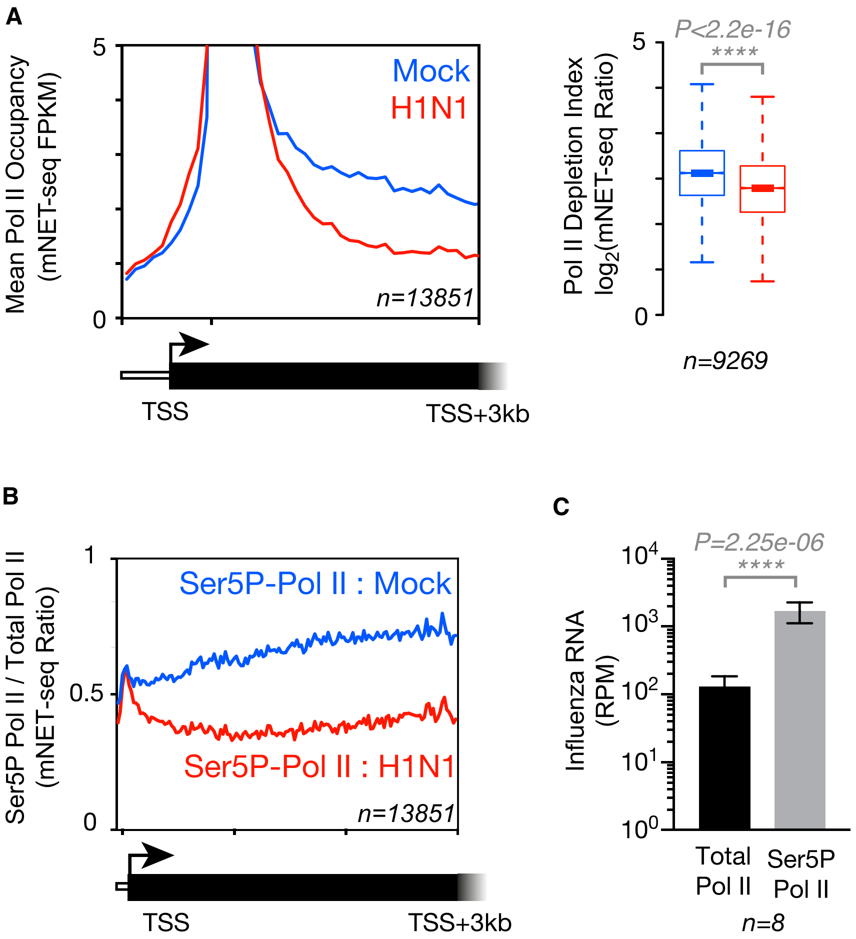

Figure 2. Influenza Virus Infection Causes Decreased Host Pol II Occupancy in Gene Bodies Downstream of Transcription Start Sites.

(A) Meta-profile of Pol II occupancy on non-overlapping protein-coding genes shows that Pol II is depleted from gene bodies downstream of transcription start sites (TSSs). Note that the y axis has been enlarged to show detail and that the full height of the profile at the TSS is shown in Figure 1B. Calculation of a Pol II depletion index (see STAR Methods) for each expressed gene and statistical analysis shows a significant depletion of Pol II in gene bodies downstream of the TSS during influenza virus infection.

(B) Meta-profile comparing the occupancy of serine 5 phosphorylated CTD isoform of Pol II (Ser5P-Pol II) with levels of total Pol II. The profiles show specific reduction of Ser5P-Pol II downstream of the TSS during influenza virus infection.

(C) Levels of the eight influenza viral RNA segments co-immunoprecipitating with Ser5P-Pol II relative to levels with total Pol II are more than 1,000-fold higher as measured by mNET-seq. This is consistent with FluPol specifically binding Ser5P-Pol II to carry out cap snatching (Lukarska et al., 2017). Error bars indicate SD among the eight viral RNA segments.

Data shown are from a single, representative biological replicate. See also Figure S2.

influenza B virus (B/Florida/04), which expresses an NS1 protein with an effector domain that bears no homology to that of the influenza A virus NS1 and does not bind to CPSF30 (Ma et al., 2016). We carried out mNET-seq on these infected cells and compared the profiles of Pol II with our previous influenza virus infections (A/WSN/33) and uninfected cells (Figures 5A, 5B, and S3). Surprisingly, we found that Pol II termination was significantly defective in all cases, irrespective of virus strain. To confirm these findings, we carried out infections and mNET-seq analysis with an H3N2 strain of influenza A virus with a strong NS1-CPSF30 interaction (A/Udorn/72) (Das et al., 2008) and with a mutant virus of the same strain in which the NS1 protein is truncated and lacks the CPSF30-binding effector domain (NS1Δ99) (Jackson et al., 2010). The wild-type H3N2 virus induces a significant termination defect, similar to that observed previously with H1N1. The mutant virus with a truncated NS1 protein causes a similar defect in Pol II termination (Figures 5C and 5D), even though its growth is restricted, and very little depletion of Pol II occurs in gene bodies. We therefore conclude that the termination defect we observe can arise from influenza virus infection alone, irrespective of direct interaction between the viral NS1 protein and CPSF30.

Osmotic Shock Induces a Pol II Termination Defect Similar to Influenza Virus Infection

We next considered whether the Pol II termination defect we observe might be similar to a more generalized cellular stress response. Osmotic shock, for example, has been reported to cause the production of downstream-of-gene transcripts (DoGs) that arise from continued transcription tens of kilobases downstream of normal poly(A) sites (Vilborg et al., 2015). These DoGs, which occur on a limited set of genes and are polyadenylated, appear to be substantially less abundant than their properly terminated counterparts in nuclear RNA poly(A)$^+$ RNA (Vilborg et al., 2015). We examined Pol II behavior directly during osmotic shock by performing mNET-seq on cells treated with 80 mM KCl for 1 hr (Figure 6A). Remarkably, osmotic shock also produces a strong termination defect (Figure 6B), and the effect we observe using mNET-seq is greater and more widespread than previously described on the basis of RNA sequencing (RNA-seq) (Vilborg et al., 2015). This termination defect, resulting from osmotic shock, appeared comparable with that resulting from influenza virus infection, so we compared the read-through index of each gene during influenza virus infection and osmotic shock and found that the extent of Pol II read-through was well correlated (Spearman's $r = 0.73$; Figure 6C). On the basis of this finding, we compared mNET-seq profiles of Pol II occupancy downstream of genes with nuclear RNA-seq profiles of DoGs generated from published data (Figure 6D) and found that DoGs match well to regions of Pol II read-through

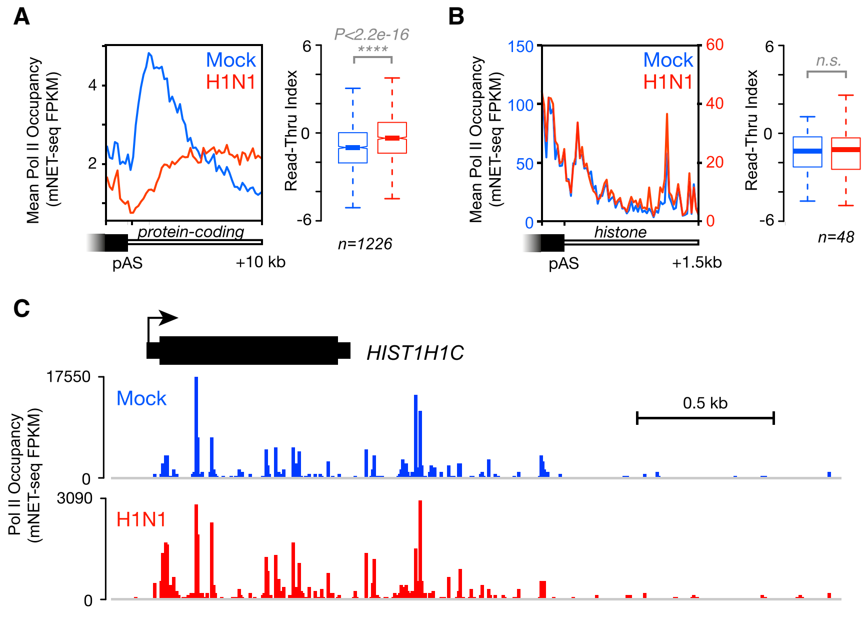

Figure 3. Pol II Fails to Terminate Downstream of Protein-Coding Genes During Influenza Virus Infection

(A) Meta-profiles of Pol II occupancy at the 3′ end of expressed protein-coding genes with a single poly(A) site as well as statistical analysis of Pol II read-through on each gene (read-through index; see STAR Methods) show that there is a significant defect in Pol II termination during influenza virus infection.

(B) Meta-profiles of Pol II occupancy at the 3′ end of histone genes and statistical analysis of histone gene read-through indices show that Pol II termination is not affected downstream of histone genes, which are processed differently to canonical mRNAs (Kolev and Steitz, 2005). Although overall levels of Pol II occupancy on histone genes drop during influenza virus infection, the pattern of Pol II behavior remains the same. Note that the meta-profile y axis scales for Mock (left, blue) and H1N1 (right, red) differ.

(C) mNET-seq profile of Pol II occupancy along a histone gene, *HIST1H1C*.

Data shown are from a single, representative biological replicate.

caused by influenza virus infection. Using RT-qPCR of total cellular RNA, we found that we could detect the production of DoGs specifically in response to both influenza virus infection and NS1 protein expression (Figure S4). Similarly, we found that the Pol II read-through indices for genes with identified DoGs are significantly higher than for genes without DoGs (Figure 6E). We conclude that DoGs produced during cellular stress likely arise from catastrophically altered termination (CAT) in which Pol II proceeds into regions that would normally remain un-transcribed. This same phenomenon can be triggered by diverse stimuli, including osmotic shock or viral infection as we show here, as well as heat shock (Mahat et al., 2016) and oxidative stress (Giannakakis et al., 2015).

DISCUSSION

We have analyzed the behavior of host Pol II during influenza virus infection and show that influenza virus executes a two-pronged attack on host transcription, with (1) FluPol association with Pol II leading to depletion of Pol II in gene bodies and (2) viral stress and viral protein-mediated interference of CPA leading to a marked termination defect at normal poly(A) sites. Our results have implications for understanding both viral effects and fundamental aspects of mammalian transcription that have consequences for virus-host interactions.

Viral Interference with Pol II Leads to Host Shutoff

Our observation that influenza virus infection leads to decreased Pol II gene occupancy downstream of the TSS suggests that FluPol association with Pol II can act as a simple mechanism of host shutoff by dysregulating host transcription. In contrast to the termination defect at the end of genes (which can be induced by viral NS1 protein or by cellular stress), this depletion of Pol II downstream of the TSS is specific to influenza virus infection. A number of mechanisms could underlie the decreased Pol II occupancy we observe. One model would involve FluPol inducing extremely premature termination of Pol II, perhaps mediated by cleavage of nascent host transcripts by FluPol that renders the exposed 5′ end of the nascent Pol II transcript a suitable substrate for degradation by Xrn2. This would lead to premature termination as a result of Pol II being "torpedoed" (Kim et al., 2004; West et al., 2004) off gene bodies during influenza virus infection. Such a mechanism, involving stochastic binding of Xrn2 to the exposed cleaved transcript, would also be consistent with the tailing-off of Pol II occupancy we observe downstream of the TSS (Figure S2). Thus, FluPol-induced termination would mimic canonical Pol II termination at the poly(A) site (pAS), in which both transcript cleavage (here performed by FluPol instead of CPSF73) and Xrn2 activity are required (Eaton et al., 2018). Such a process is also reminiscent of Nrd1-mediated termination in yeast (Vasiljeva et al., 2008) and previous results showing that mRNA decapping in mammals leads to Pol II termination via Xrn2 action (Brannan et al., 2012).

A second model would involve FluPol blocking access of Pol II regulatory factors to Pol II and its CTD (Jonkers and Lis, 2015). Such a process could involve the inhibition of productive Pol II elongation, for example by preventing P-TEFb-dependent phosphorylation of Ser2 of the Pol II CTD, leading to more promoter-proximal pausing (Zaborowska et al., 2016b). Our mNET-seq data, however, show similar initial peaks of paused Pol II levels downstream of the TSS before the signal decreases in the gene body (Figures 1B, 2B, and S2). Alternatively, the presence of FluPol could result in an increase in the elongation rate of Pol II (and consequently a decrease in observed mNET-seq Pol II occupancy), for example by preventing the binding of repressive factors or by increasing the recruitment of processivity factors such as elongin (Kwak and Lis, 2013).

In both cases, dysregulation of Pol II transcription downstream of the TSS will have functional consequences for host

OPEN
ACCESS
CellPress

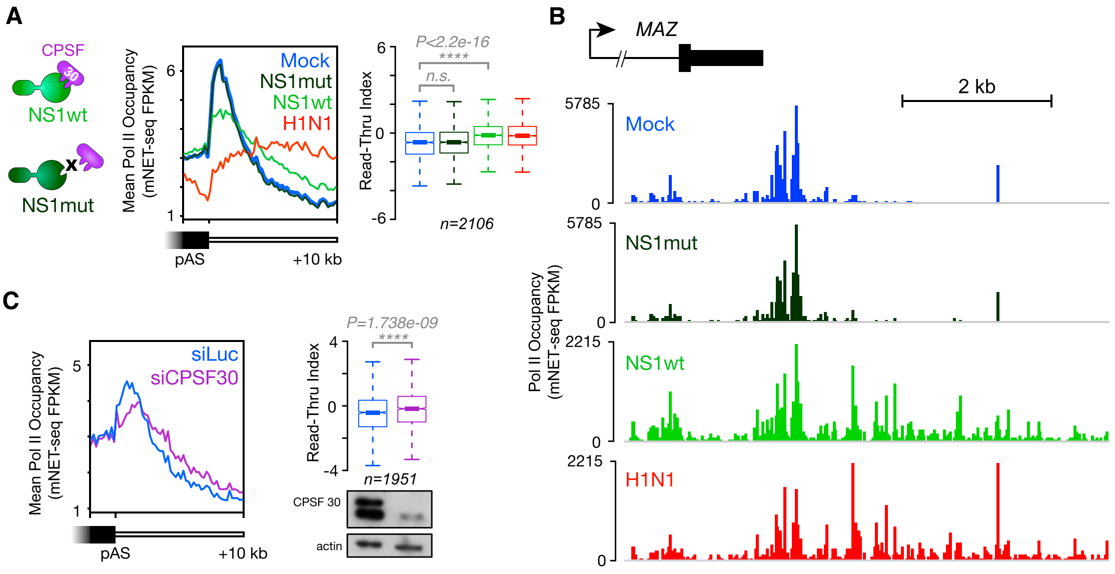

Figure 4. The Influenza Virus NS1 Protein Induces a Host Pol II Termination Defect

(A) Induced expression of the viral NS1 protein (NS1wt) or a CPSF30-binding mutant (NS1mut) in HEK293 cells was compared with influenza virus infection (H1N1) or uninduced and uninfected cells (Mock). Meta-profiles of Pol II occupancy at the 3′ end of expressed protein-coding genes with a single poly(A) site as well as statistical analysis of Pol II read-through on each gene reveal that the viral NS1 protein alone induces a Pol II termination defect downstream of poly(A) sites. Note that in contrast to viral infection, NS1 expression does not deplete Pol II in gene bodies, as reflected by the difference in baseline Pol II occupancy prior to the poly(A) site.

(B) mNET-seq profiles of Pol II occupancy at the 3′ end of a protein-coding gene, *MAZ*. The unexpressed downstream *PRRT2* gene is not shown in the annotation.

(C) siRNA knockdown of CPSF30 also produces a Pol II termination defect similar to NS1 protein expression. The meta-profiles of Pol II occupancy at the 3′ end of expressed protein-coding genes with a single poly(A) site are shown, as well as statistical analysis of Pol II read-through on each gene are shown, and a western blot (see STAR Methods) confirming successful CPSF30 knockdown.

Data shown are from a single, representative biological replicate.

shutoff. In the context of a cell in which antiviral gene expression has been triggered, the recruitment of Pol II to these genes will also consequently recruit FluPol, allowing the virus to counteract the activation of cellular defenses. Any subsequent extremely premature Pol II termination that occurs as a result of cap snatching could also benefit viral transcription in cases where Pol II is more rapidly cleared from promoters and gene bodies, because free Pol II would now be free to re-initiate another round of transcription to feed further capped nascent RNA to FluPol. Such a relationship between apparent Pol II depletion in gene bodies and host shutoff is consistent with observations that most infected cells fail to induce a detectable interferon response even in the absence of NS1 (Kallfass et al., 2013; Killip et al., 2015, 2017; Russell et al., 2018). It seems plausible that FluPol association with Pol II could itself contribute to host shutoff, independently of any interference with host mRNA 3′ end processing.

Although it appears advantageous for a virus such as influenza to prevent expression of antiviral genes by interfering with CPA, it is also possible that failure to disengage Pol II at the end of genes during influenza virus infection provides a benefit to the host cell. Thus, continued transcription by Pol II might decrease the pool of Pol II available to re-initiate transcription and thereby starve influenza virus of the nascent, capped transcripts it needs to prime its own viral mRNA production. Interestingly, analysis of circulating H1N1 viruses suggests that CPSF30 inhibition is advantageous during adaptation to human (but not necessarily other mammalian) hosts (Clark et al., 2017). Additional work will be needed to understand how interference with host transcript CPA and its effects on global Pol II behavior affect viral function, adaptation, and evolution.

Structural analysis of how host shutoff operates is also lacking. Although we know that FluPol binds the Pol II CTD (Lukarska et al., 2017), how FluPol, in the context of vRNPs, associates with the host transcription complex at the start of genes (Hantsche and Cramer, 2017) and accommodates the capping enzyme (Martinez-Rucobo et al., 2015) has yet to be structurally characterized. Such work will be key to further evaluating the mechanisms by which influenza virus infection causes dysregulation of Pol II downstream of the TSS. Similarly, crystal structures of the effector domain of NS1 in complex with zinc fingers 2 and 3 of CPSF30 suggest that NS1 depletes CPSF30 from the CPA complex (Das et al., 2008). However, recent proteomics analysis of the influenza protein interactome has shown that NS1 from CPSF30-binding influenza virus strains is also in complex with other CPSF subunits (Wang et al., 2017). Future work to understand how both the RNA-binding domain and the effector domain of NS1, possibly in association with FluPol (Kuo and Krug, 2009), interact with the CPSF complex (Casañal et al., 2017; Clerici et al., 2017, 2018; Sun et al., 2018) will provide insights into the molecular mechanisms of viral interference with the wider CPA machinery.

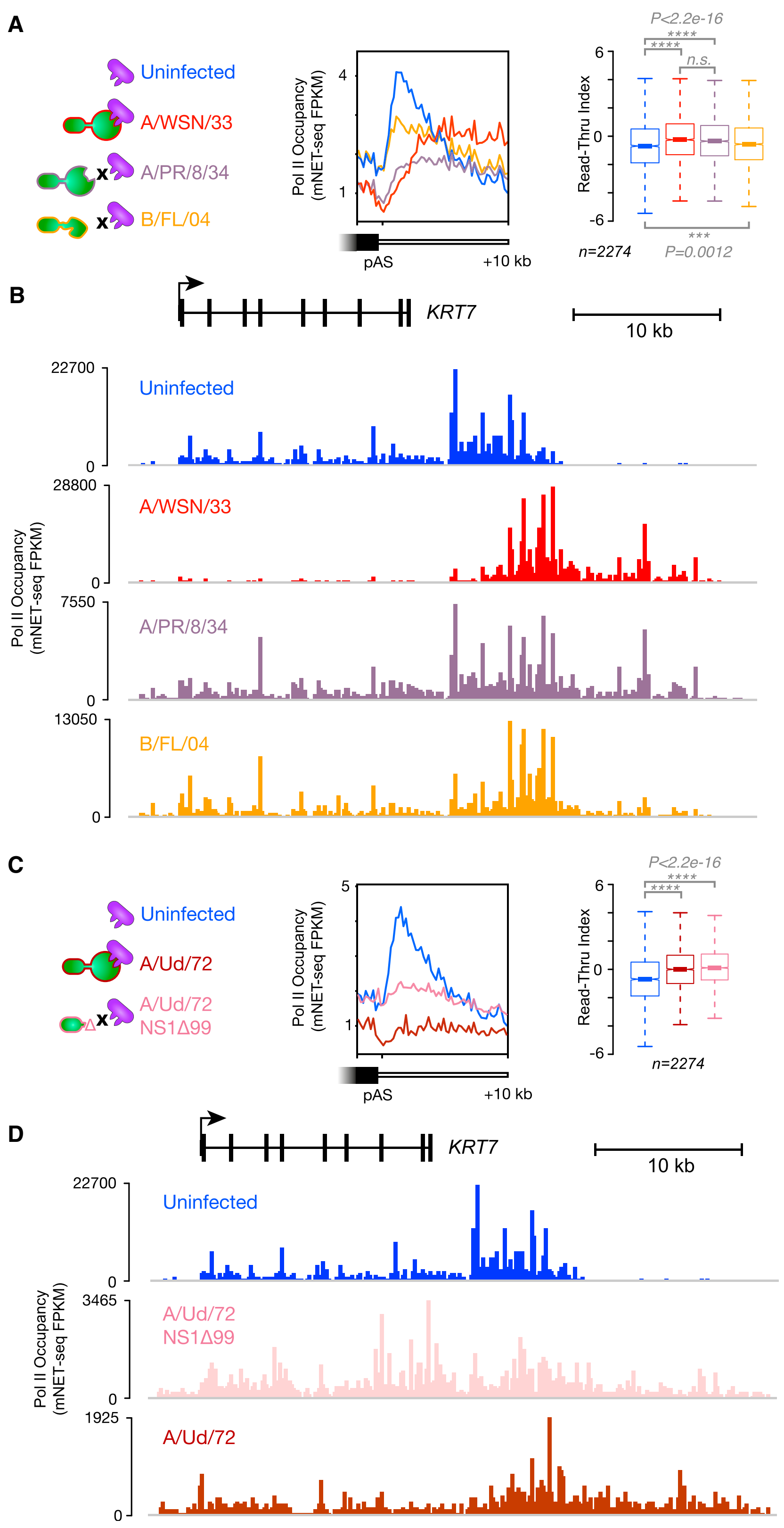

Figure 5. Influenza Virus Infection Causes a Host Pol II Termination Defect Independently of NS1-CPSF30 Interaction

(A) The closely related H1N1 strains (A/WSN/33 and A/PR/8/34) encode NS1 proteins that differ in the ability of their C-terminal effector domains to bind CPSF30. Evolutionarily distinct influenza B viruses, including B/Florida/04/2006 (B/FL/04), encode an unrelated effector domain that does not bind CPSF30. Meta-profiles of Pol II occupancy at the 3′ end of expressed protein-coding genes with a single poly(A) site as well as statistical analysis of Pol II read-through on each gene during viral infection show that all three viruses induce a significant failure of Pol II to terminate downstream of poly(A) sites.

(B) mNET-seq profiles of Pol II occupancy at the 3′ end of a protein-coding gene, *KRT7*. The unexpressed downstream gene, *KRT87P*, is not shown in the annotation.

(C) The H3N2 influenza virus (A/Udorn/72) encodes an NS1 protein with an effector domain that binds CPSF30. Infections were performed in parallel with a mutant virus of the same strain in which the NS1 protein is truncated to remove the effector domain (NS1Δ99). Meta-profiles of Pol II occupancy at the 3′ end of expressed protein-coding genes with a single poly(A) site as well as statistical analysis of Pol II read-through on each gene during viral infection show that both the wild-type and the mutant viruses induce a strong termination defect.

(D) mNET-seq profiles of Pol II occupancy during H3N2 viral infection at the 3′ end of a protein-coding gene, *KRT7*. The unexpressed downstream gene, *KRT87P*, is not shown in the annotation.

Data shown are from a single mNET-seq biological replicate. See also Figure S3.

OPEN
ACCESS
CellPress

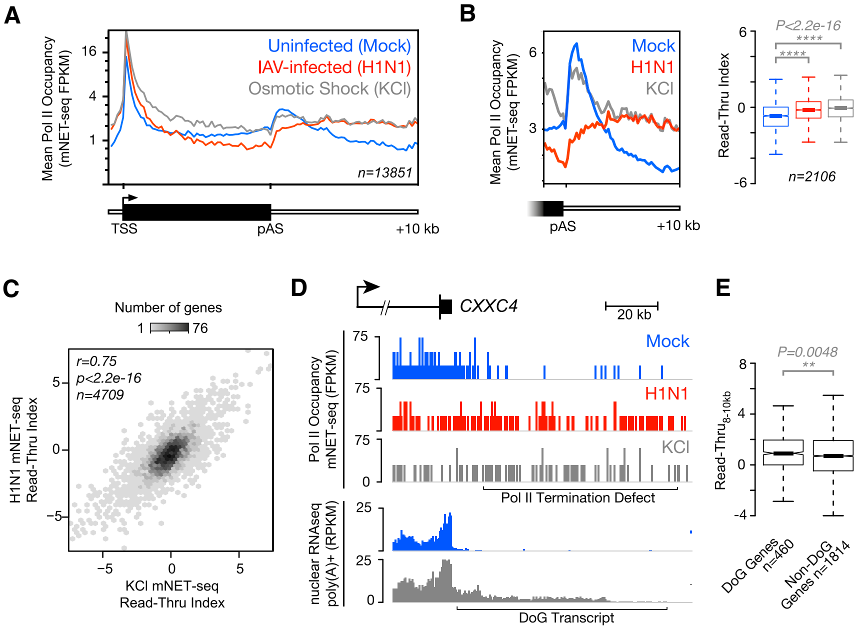

Figure 6. Pol II Termination Defects During Influenza Virus Infection and Osmotic Shock Are Linked to Downstream-of-Gene Transcript Production

(A) Meta-profiles of Pol II occupancy on all non-overlapping protein-coding genes (n = 13,851) comparing influenza virus infection and osmotic shock. Osmotic shock does not cause Pol II to be depleted in gene bodies, but it causes a failure of Pol II to terminate downstream of the poly(A) site that appears markedly similar to that during influenza virus infection.

(B) Meta-profiles of Pol II occupancy at the 3′ end of expressed protein-coding genes with a single poly(A) site as well as statistical analysis of Pol II read-through on each gene demonstrate that both osmotic shock and influenza virus infection cause a significant amount of read-through transcription.

(C) Comparison of the read-through index of each gene during influenza virus infection to the read-through index of each gene during osmotic shock shows that the two indices are correlated (Pearson's r = 0.75).

(D) mNET-seq profiles of Pol II occupancy at the 3′ end of the previously characterized (Vilborg et al., 2015) DoG-producing gene *CXXC4* show that regions downstream of genes where Pol II fails to terminate during viral infection and osmotic shock overlap with regions of DoG production. DoGs are produced during influenza virus infection (see Figure S4).

(E) Genes previously reported to produce DoGs during osmotic shock tend to have a greater extent of Pol II read-through during influenza virus infection, reflected in a significant difference in their read-through indices 8–10 kb downstream of their poly(A) site.

Data shown are from a single, representative biological replicate. See also Figure S4.

Viral Interference with Pol II Reveals Fundamental Features of Host Transcription Termination

We observe multiple effects of influenza virus infection on the behavior of Pol II. First, Pol II is depleted downstream of TSSs as a consequence of influenza virus infection. Intriguingly, although Pol II termination downstream of poly(A) sites usually results in an accumulation of paused Pol II (and thus an increased mNET-seq signal) (Nojima et al., 2015; Proudfoot, 2016), the lack of any apparent stalled Pol II downstream of TSSs during influenza virus infection suggests that any premature termination would occur either more rapidly than canonical termination downstream of poly(A) sites (e.g., because of the absence of sequence-encoded pauses normally found downstream of the poly(A) site) or more stochastically (e.g., because of a lack of coordination between transcript cleavage and premature Pol II termination). Future work will examine the dependence of influenza virus infection on the function of factors involved in Pol II termination (Proudfoot, 2016), as well as the role of the CPA complex in this extremely premature termination process.

Second, we observe widespread failure of Pol II to terminate downstream of poly(A) sites during both influenza virus infection and cellular stress from osmotic shock. These results suggest that previously observed DoGs arise from CAT. Although the DoGs that have been observed and characterized are polyadenylated and appear downstream of only a subset of genes (Vilborg et al., 2015), the Pol II termination defect caused by influenza virus infection and osmotic stress appears widespread. It is likely that DoGs are universally produced by Pol II but that only those released from chromatin are detected by conventional RNA-seq. This is consistent with the observation that DoGs are less likely to contain a consensus poly(A) signal in their body (Vilborg et al., 2017). DoGs might escape when Pol II encounters a feature, such as a cryptic poly(A) site or a secondary structure-rich region (e.g., facilitating backtracking or R-loop formation) (Proudfoot, 2016), which might then promote transcript release or allow it to escape degradation by the nuclear exosome. Although the production of DoGs may simply be incidental, it is plausible that some transcripts have evolved to serve a function.

The observed Pol II termination defect during influenza virus infection could restrict the amount capped nascent RNA available to FluPol and therefore be a protective cellular response. However, viral infection might simply be causing general cellular stress conditions that induce a Pol II termination defect or affect pathways related to these conditions. Influenza virus is known to induce oscillations in calcium ion concentrations (Fujioka et al., 2013), which might trigger an osmotic shock response. Similarly, viral activation of inflammation (Tavares et al., 2017) could generate reactive oxygen species (ROS). Also, RIG-I binding to the mitochondrial antiviral-signaling protein (MAVS) (Hou et al., 2011; Killip et al., 2015) might allow ROS release from the mitochondria (Koshiba, 2013) to trigger an oxidative stress response. Last, heat shock proteins and pathways intersect with various aspects of influenza virus infection. For example, Hsp90 is reported to be hijacked for the nuclear import of the viral polymerase subunits (Naito et al., 2007), and SUMO remodeling in response to heat shock is reminiscent of the changes in host SUMOylation induced during influenza virus infection (Domingues et al., 2015).

It is also possible that the influenza virus-induced termination defect relates to a more general stress response, for example to maintain chromatin structural integrity (Vilborg et al., 2015) during osmotic or heat shock. A similar role in the preservation of genomic integrity might also be at play during oxidative stress, whereby Pol II may be pressed into acting as a sentry for oxidative lesions or ssDNA breaks, with the termination defect allowing Pol II to travel into normally inaccessible regions of the chromatin. More broadly, the question remains as to why the general cellular response to a wide variety of ‘‘stresses’’ appears to be the dysregulation of CPA and Pol II termination. Might there be a simple reason underlying this common phenotype? A possible explanation is that prevention of transcript CPA is a fail-safe mechanism to eliminate now unwanted transcripts arising from already elongating Pol II. We expect that future work on both cellular stress and influenza virus infection will further inform our understandings of the mechanism and coordination of transcription termination.

STAR★METHODS

Detailed methods are provided in the online version of this paper and include the following:

- KEY RESOURCES TABLE
- CONTACT FOR REAGENT AND RESOURCE SHARING
- EXPERIMENTAL MODEL AND SUBJECT DETAILS
- METHOD DETAILS
  - Infection, Expression, and Osmotic Shock Experiments
  - Sample Preparation for mNET-seq Experiments
  - Library Preparation and Sequencing for mNET-seq Experiments
  - Data Processing for mNET-seq
- QUANTIFICATION AND STATISTICAL ANALYSIS
- DATA AND SOFTWARE AVAILABILITY

SUPPLEMENTAL INFORMATION

Supplemental Information includes four figures and two tables and can be found with this article online at https://doi.org/10.1016/j.celrep.2018.04.047.

ACKNOWLEDGMENTS

We thank Wendy Barclay, Rebecca Frise, and Marian Killip for providing viruses and Steven West for providing cell lines. We thank Joan Steitz and Lars Dölken for helpful discussions, comments, and sharing data before publication. We are also grateful to Justyna Zaborowska, Kinga Kamieniarz-Gdula, and the E.F., N.J.P., and S.M. labs for helpful advice. This work was funded by Medical Research Council (MRC) programme grant MR/K000241/1 (to E.F.), an EPA Cephalosporin Junior Research Fellowship (to D.L.V.B.), and a Marie Curie Intra European Fellowship within the 7th European Community Framework Programme PIEF-GA-2012-32§8746 (to M.M.-A.). This work was also supported by European Research Council advanced grant 339270 (to N.J.P.) and Wellcome Trust Investigator awards 107928/Z/15/Z (to N.J.P.) and WT106134A (to S.M.). We thank the High-Throughput Genomics Group at the Wellcome Trust Centre for Human Genetics (funded by Wellcome Trust grant 090532/Z/09/Z) for the generation of the sequencing data.

AUTHOR CONTRIBUTIONS

D.L.V.B., M.M.-A., N.J.P., and E.F. designed research. D.L.V.B., M.M.-A., and T.N. performed research. D.L.V.B. and M.T. analyzed data. N.J.P., S.M., and E.F. supervised research and analysis. D.L.V.B. and E.F. wrote the paper.

DECLARATION OF INTERESTS

The authors declare no competing interests.

Received: February 3, 2018
Revised: April 3, 2018
Accepted: April 11, 2018
Published: May 15, 2018

REFERENCES

Bercovich-Kinori, A., Tai, J., Gelbart, I.A., Shitrit, A., Ben-Moshe, S., Drori, Y., Itzkovitz, S., Mandelboim, M., and Stern-Ginossar, N. (2016). A systematic view on influenza induced host shutoff. eLife *5*, 600.

Brannan, K., Kim, H., Erickson, B., Glover-Cutter, K., Kim, S., Fong, N., Kiemele, L., Hansen, K., Davis, R., Lykke-Andersen, J., and Bentley, D.L. (2012). mRNA decapping factors and the exonuclease Xrn2 function in widespread premature termination of RNA polymerase II transcription. Mol. Cell *46*, 311–324.

Buratowski, S. (2005). Connections between mRNA 3′ end processing and transcription termination. Curr. Opin. Cell Biol. *17*, 257–261.

Casañal, A., Kumar, A., Hill, C.H., Easter, A.D., Emsley, P., Degliesposti, G., Gordiyenko, Y., Santhanam, B., Wolf, J., Wiederhold, K., et al. (2017). Architecture of eukaryotic mRNA 3′-end processing machinery. Science *358*, 1056–1059.

Chan, A.Y., Vreede, F.T., Smith, M., Engelhardt, O.G., and Fodor, E. (2006). Influenza virus inhibits RNA polymerase II elongation. Virology *351*, 210–217.

Clark, A.M., Nogales, A., Martinez-Sobrido, L., Topham, D.J., and DeDiego, M.L. (2017). Functional evolution of influenza virus NS1 protein in currently circulating human 2009 pandemic H1N1 viruses. J. Virol. *91*, e00721–17.

Clerici, M., Faini, M., Aebersold, R., and Jinek, M. (2017). Structural insights into the assembly and polyA signal recognition mechanism of the human CPSF complex. eLife *6*, 213.

Clerici, M., Faini, M., Muckenfuss, L.M., Aebersold, R., and Jinek, M. (2018). Structural basis of AAUAAA polyadenylation signal recognition by the human CPSF complex. Nat. Struct. Mol. Biol. *263*, 211.

Das, K., Ma, L.-C., Xiao, R., Radvansky, B., Aramini, J., Zhao, L., Marklund, J., Kuo, R.-L., Twu, K.Y., Arnold, E., et al. (2008). Structural basis for suppression of a host antiviral response by influenza A virus. Proc. Natl. Acad. Sci. U S A *105*, 13093–13098.

Davidson, L., Muniz, L., and West, S. (2014). 3′ end formation of pre-mRNA and phosphorylation of Ser2 on the RNA polymerase II CTD are reciprocally coupled in human cells. Genes Dev. *28*, 342–356.

Dobin, A., Davis, C.A., Schlesinger, F., Drenkow, J., Zaleski, C., Jha, S., Batut, P., Chaisson, M., and Gingeras, T.R. (2013). STAR: ultrafast universal RNA-seq aligner. Bioinformatics *29*, 15–21.

Domingues, P., Golebiowski, F., Tatham, M.H., Lopes, A.M., Taggart, A., Hay, R.T., and Hale, B.G. (2015). Global reprogramming of host SUMOylation during influenza virus infection. Cell Rep. *13*, 1467–1480.

Eaton, J., Davidson, L., Bauer, D.L., Natsume, T., Kanemaki, M.T., West, S., and West, J.S. (2018). Xrn2 accelerates termination by RNA polymerase II, which is underpinned by CPSF73 activity. Genes Dev. *32*, 127–139.

Eisfeld, A.J., Neumann, G., and Kawaoka, Y. (2015). At the centre: influenza A virus ribonucleoproteins. Nat. Rev. Microbiol. *13*, 28–41.

Engelhardt, O.G., Smith, M., and Fodor, E. (2005). Association of the influenza A virus RNA-dependent RNA polymerase with cellular RNA polymerase II. J. Virol. *79*, 5812–5818.

Fodor, E., Devenish, L., Engelhardt, O.G., Palese, P., Brownlee, G.G., and García-Sastre, A. (1999). Rescue of influenza A virus from recombinant DNA. J. Virol. *73*, 9679–9682.

Fujioka, Y., Tsuda, M., Nanbo, A., Hattori, T., Sasaki, J., Sasaki, T., Miyazaki, T., and Ohba, Y. (2013). A Ca(2+)-dependent signalling circuit regulates influenza A virus internalization and infection. Nat. Commun. *4*, 2763.

Giannakakis, A., Zhang, J., Jenjaroenpun, P., Nama, S., Zainolabidin, N., Aau, M.Y., Yarmishyn, A.A., Vaz, C., Ivshina, A.V., Grinchuk, O.V., et al. (2015). Contrasting expression patterns of coding and noncoding parts of the human genome upon oxidative stress. Sci. Rep. *5*, 9737.

Glover-Cutter, K., Kim, S., Espinosa, J., and Bentley, D.L. (2008). RNA polymerase II pauses and associates with pre-mRNA processing factors at both ends of genes. Nat. Struct. Mol. Biol *15*, 71–78.

Hale, B.G., Steel, J., Medina, R.A., Manicassamy, B., Ye, J., Hickman, D., Hai, R., Schmolke, M., Lowen, A.C., Perez, D.R., and García-Sastre, A. (2010). Inefficient control of host gene expression by the 2009 pandemic H1N1 influenza A virus NS1 protein. J. Virol. *84*, 6909–6922.

Hantsche, M., and Cramer, P. (2017). Conserved RNA polymerase II initiation complex structure. Curr. Opin. Struct. Biol. *47*, 17–22.

Harrow, J., Frankish, A., Gonzalez, J.M., Tapanari, E., Diekhans, M., Kokocinski, F., Aken, B.L., Barrell, D., Zadissa, A., Searle, S., et al. (2012). GENCODE: the reference human genome annotation for The ENCODE Project. Genome Res. *22*, 1760–1774.

Herbert, K.M., and Nag, A. (2016). A tale of two RNAs during Viral Infection: How Viruses Antagonize mRNAs and small non-coding RNAs in the host cell. Viruses *8*, 154.

Ho, C.K., and Shuman, S. (1999). Distinct roles for CTD Ser-2 and Ser-5 phosphorylation in the recruitment and allosteric activation of mammalian mRNA capping enzyme. Mol. Cell *3*, 405–411.

Hou, F., Sun, L., Zheng, H., Skaug, B., Jiang, Q.-X., and Chen, Z.J. (2011). MAVS forms functional prion-like aggregates to activate and propagate antiviral innate immune response. Cell *146*, 448–461.

Jackson, D., Killip, M.J., Galloway, C.S., Russell, R.J., and Randall, R.E. (2010). Loss of function of the influenza A virus NS1 protein promotes apoptosis but this is not due to a failure to activate phosphatidylinositol 3-kinase (PI3K). Virology *396*, 94–105.

Jackson, D., Elderfield, R.A., and Barclay, W.S. (2011). Molecular studies of influenza B virus in the reverse genetics era. J. Gen. Virol. *92*, 1–17.

Jonkers, I., and Lis, J.T. (2015). Getting up to speed with transcription elongation by RNA polymerase II. Nat. Rev. Mol. Cell Biol. *16*, 167–177.

Kallfass, C., Lienenklaus, S., Weiss, S., and Staeheli, P. (2013). Visualizing the beta interferon response in mice during infection with influenza A viruses expressing or lacking nonstructural protein 1. J. Virol. *87*, 6925–6930.

Killip, M.J., Fodor, E., and Randall, R.E. (2015). Influenza virus activation of the interferon system. Virus Res. *209*, 11–22.

Killip, M.J., Jackson, D., Pérez-Cidoncha, M., Fodor, E., and Randall, R.E. (2017). Single-cell studies of IFN-β promoter activation by wildtype and NS1-defective influenza A viruses. J. Gen. Virol. *98*, 357–363.

Kim, M., Krogan, N.J., Vasiljeva, L., Rando, O.J., Nedea, E., Greenblatt, J.F., and Buratowski, S. (2004). The yeast Rat1 exonuclease promotes transcription termination by RNA polymerase II. Nature *432*, 517–522.

Kolev, N.G., and Steitz, J.A. (2005). Symplekin and multiple other polyadenylation factors participate in 3′-end maturation of histone mRNAs. Genes Dev. *19*, 2583–2592.

Koshiba, T. (2013). Mitochondrial-mediated antiviral immunity. Biochim. Biophys. Acta *1833*, 225–232.

Krug, R.M., Broni, B.A., and Bouloy, M. (1979). Are the 5′ ends of influenza viral mRNAs synthesized in vivo donated by host mRNAs? Cell *18*, 329–334.

Kuo, R.-L., and Krug, R.M. (2009). Influenza a virus polymerase is an integral component of the CPSF30-NS1A protein complex in infected cells. J. Virol. *83*, 1611–1616.

Kwak, H., and Lis, J.T. (2013). Control of transcriptional elongation. Annu. Rev. Genet. *47*, 483–508.

Lukarska, M., Fournier, G., Pflug, A., Resa-Infante, P., Reich, S., Naffakh, N., and Cusack, S. (2017). Structural basis of an essential interaction between influenza polymerase and Pol II CTD. Nature *541*, 117–121.

Ma, L.-C., Guan, R., Hamilton, K., Aramini, J.M., Mao, L., Wang, S., Krug, R.M., and Montelione, G.T. (2016). A second RNA-binding site in the NS1 protein of influenza B virus. Structure *24*, 1562–1572.

Mahat, D.B., Salamanca, H.H., Duarte, F.M., Danko, C.G., and Lis, J.T. (2016). Mammalian heat shock response and mechanisms underlying its genome-wide transcriptional regulation. Mol. Cell *62*, 63–78.

Martin, M. (2011). Cutadapt removes adapter sequences from high-throughput sequencing reads. EMBnet.journal *17*, 10.

Martínez-Alonso, M., Hengrung, N., and Fodor, E. (2016). RNA-free and ribonucleoprotein-associated influenza virus polymerases directly bind the serine-5-phosphorylated carboxyl-terminal domain of host RNA polymerase II. J. Virol. *90*, 6014–6021.

Martinez-Rucobo, F.W., Kohler, R., van de Waterbeemd, M., Heck, A.J.R., Hemann, M., Herzog, F., Stark, H., and Cramer, P. (2015). Molecular basis of transcription-coupled pre-mRNA capping. Mol. Cell *58*, 1079–1089.

Naito, T., Momose, F., Kawaguchi, A., and Nagata, K. (2007). Involvement of Hsp90 in assembly and nuclear import of influenza virus RNA polymerase subunits. J. Virol. *81*, 1339–1349.

Nemeroff, M.E., Barabino, S.M., Li, Y., Keller, W., and Krug, R.M. (1998). Influenza virus NS1 protein interacts with the cellular 30 kDa subunit of CPSF and inhibits 3’end formation of cellular pre-mRNAs. Mol. Cell *1*, 991–1000.

Neve, J., Burger, K., Li, W., Hoque, M., Patel, R., Tian, B., Gullerova, M., and Furger, A. (2016). Subcellular RNA profiling links splicing and nuclear DICER1 to alternative cleavage and polyadenylation. Genome Res. *26*, 24–35.

Nojima, T., Gomes, T., Grosso, A.R.F., Kimura, H., Dye, M.J., Dhir, S., Carmo-Fonseca, M., and Proudfoot, N.J. (2015). Mammalian NET-seq reveals genome-wide nascent transcription coupled to RNA processing. Cell *161*, 526–540.

Nojima, T., Gomes, T., Carmo-Fonseca, M., and Proudfoot, N.J. (2016). Mammalian NET-seq analysis defines nascent RNA profiles and associated RNA processing genome-wide. Nat. Protoc. *11*, 413–428.

Poon, L.L., Pritlove, D.C., Fodor, E., and Brownlee, G.G. (1999). Direct evidence that the poly(A) tail of influenza A virus mRNA is synthesized by reiterative copying of a U track in the virion RNA template. J. Virol. *73*, 3473–3476.

Proudfoot, N.J. (2016). Transcriptional termination in mammals: Stopping the RNA polymerase II juggernaut. Science *352*, aad9926.

Ramírez, F., Dündar, F., Diehl, S., Grüning, B.A., and Manke, T. (2014). deepTools: a flexible platform for exploring deep-sequencing data. Nucleic Acids Res. *42*, W187–W191.

Richard, P., and Manley, J.L. (2009). Transcription termination by nuclear RNA polymerases. Genes Dev. *23*, 1247–1269.

Robinson, J.T., Thorvaldsdóttir, H., Winckler, W., Guttman, M., Lander, E.S., Getz, G., and Mesirov, J.P. (2011). Integrative genomics viewer. Nat. Biotechnol. *29*, 24–26.

Rodriguez, A., Pérez-González, A., and Nieto, A. (2007). Influenza virus infection causes specific degradation of the largest subunit of cellular RNA polymerase II. J. Virol. *81*, 5315–5324.

Russell, A.B., Trapnell, C., and Bloom, J.D. (2018). Extreme heterogeneity of influenza virus infection in single cells. eLife *7*, e02020.

Rutkowski, A.J., Erhard, F., L’Hernault, A., Bonfert, T., Schilhabel, M., Crump, C., Rosenstiel, P., Efstathiou, S., Zimmer, R., Friedel, C.C., and Dölken, L. (2015). Widespread disruption of host transcription termination in HSV-1 infection. Nat. Commun. *6*, 7126.

Schlackow, M., Nojima, T., Gomes, T., Dhir, A., Carmo-Fonseca, M., and Proudfoot, N.J. (2017). Distinctive patterns of transcription and RNA processing for human lincRNAs. Mol. Cell *65*, 25–38.

Subbarao, K., Chen, H., Swayne, D., Mingay, L., Fodor, E., Brownlee, G., Xu, X., Lu, X., Katz, J., Cox, N., and Matsuoka, Y. (2003). Evaluation of a genetically modified reassortant H5N1 influenza A virus vaccine candidate generated by plasmid-based reverse genetics. Virology *305*, 192–200.

Sun, Y., Zhang, Y., Hamilton, K., Manley, J.L., Shi, Y., Walz, T., and Tong, L. (2018). Molecular basis for the recognition of the human AAUAAA polyadenylation signal. Proc. Natl. Acad. Sci. U S A *115*, E1419–E1428.

Taubenberger, J.K., and Kash, J.C. (2010). Influenza virus evolution, host adaptation, and pandemic formation. Cell Host Microbe *7*, 440–451.

Tavares, L.P., Teixeira, M.M., and Garcia, C.C. (2017). The inflammatory response triggered by Influenza virus: a two edged sword. Inflamm. Res. *66*, 283–302.

te Velthuis, A.J.W., and Fodor, E. (2016). Influenza virus RNA polymerase: insights into the mechanisms of viral RNA synthesis. Nat. Rev. Microbiol. *14*, 479–493.

Vasiljeva, L., Kim, M., Mutschler, H., Buratowski, S., and Meinhart, A. (2008). The Nrd1-Nab3-Sen1 termination complex interacts with the Ser5-phosphorylated RNA polymerase II C-terminal domain. Nat. Struct. Mol. Biol. *15*, 795–804.

Vilborg, A., and Steitz, J.A. (2017). Readthrough transcription: how are DoGs made and what do they do? RNA Biol. *14*, 632–636.

Vilborg, A., Passarelli, M.C., Yario, T.A., Tycowski, K.T., and Steitz, J.A. (2015). Widespread inducible transcription downstream of human genes. Mol. Cell *59*, 449–461.

Vilborg, A., Sabath, N., Wiesel, Y., Nathans, J., Levy-Adam, F., Yario, T.A., Steitz, J.A., and Shalgi, R. (2017). Comparative analysis reveals genomic features of stress-induced transcriptional readthrough. Proc. Natl. Acad. Sci. U S A *114*, E8362–E8371.

Vreede, F.T., and Fodor, E. (2010). The role of the influenza virus RNA polymerase in host shut-off. Virulence *1*, 436–439.

Vreede, F.T., Chan, A.Y., Sharps, J., and Fodor, E. (2010). Mechanisms and functional implications of the degradation of host RNA polymerase II in influenza virus infected cells. Virology *396*, 125–134.

Wang, L., Fu, B., Li, W., Patil, G., Liu, L., Dorf, M.E., and Li, S. (2017). Comparative influenza protein interactomes identify the role of plakophilin 2 in virus restriction. Nat. Commun. *8*, 13876.

West, S., Gromak, N., and Proudfoot, N.J. (2004). Human 5′–> 3′ exonuclease Xrn2 promotes transcription termination at co-transcriptional cleavage sites. Nature *432*, 522–525.

Zaborowska, J., Egloff, S., and Murphy, S. (2016a). The pol II CTD: new twists in the tail. Nat. Struct. Mol. Biol. *23*, 771–777.

Zaborowska, J., Isa, N.F., and Murphy, S. (2016b). P-TEFb goes viral. Inside Cell *1*, 106–116.

STAR★METHODS

KEY RESOURCES TABLE

REAGENT or RESOURCE	SOURCE	IDENTIFIER
Antibodies		
Mouse IgG1 Anti-Pol II CTD mAb	MBL Life Science	MABI0601
Anti-Phospho Pol II CTD (Ser5) mAb	MBL Life Science	MABI0603
Anti-Actin antibody produced in rabbit	Sigma	A2066 RRID:AB_476693
Rabbit anti-CPSF30 Antibody, Affinity Purified	Bethyl Laboratories	A301-584A RRID:AB_1078872
Bacterial and Virus Strains		
H1N1 Influenza Virus, A/WSN/33	(Fodor et al., 1999)	WSN
H1N1 Influenza Virus, A/Puerto Rico/8/34	(Subbarao et al., 2003)	PR8
H3N2 Influenza Virus, A/Udorn/72	(Jackson et al., 2010)	Ud
Influenza B Virus, B/Florida/04/2006	(Jackson et al., 2011)	B/FL
H3N2 Influenza Virus, A/Udorn/72: NS1Δ99	(Jackson et al., 2010)	UdDel99
Chemicals, Peptides, and Recombinant Proteins		
RNAiMAX transfection reagent	Invitrogen	13778-150
Reagents for mNET-seq sample preparation	(Nojima et al., 2016)	See paper for details
Critical Commercial Assays		
Brilliant III Ultra-Fast SYBR Green QPCR Master Mix	Agilent	600889
Reagents for mNET-seq sample preparation	(Nojima et al., 2016)	See paper for details
Quick-RNA microprep kit	Zymo Research	R1050
TruSeq Small RNA Library Preparation kit	Illumina	RS-200-0012
NEBNext Small RNA Library Preparation kit	NEB	E7300
Bioanalyzer Agilent High Sensitivity DNA Kit	Agilent	5067-4626
Qubit dsDNA HS Assay Kit	Invitrogen	Q32851
Deposited Data		
Raw sequencing data	This paper; NCBI SRA	SRP132032
Poly(A) Site usage	(Neve et al., 2016)	See source SI
Lists of DoG transcript genes during osmotic shock	(Vilborg et al., 2015)	See source SI
Experimental Models: Cell Lines		
Human adenocarcinoma alveolar basal epithelial cells (A549)	ATCC	A549 RRID:CVCL_0023
Madin-Darby Bovine Kidney cells (MDBK)	ATCC	MDBK RRID:CVCL_0421
Madin-Darby Canine Kidney cells (MDCK)	ATCC	MDCK RRID:CVCL_0422
Human embryonic kidney 293 Flp-In TRex: NS1wt	(Davidson et al., 2014)	NS1wt
Human embryonic kidney 293 Flp-In TRex: NS1mut	(Davidson et al., 2014)	NS1mut
Oligonucleotides		
Control siRNA (siLuc) Sense (5′-3′): GAUUAUGUCCGGUUAUGUAUU Antisense (5′-3′): [p]UACAUAACCGGACAUAAUCUU	(Nojima et al., 2015)	siLuc
SMARTpool ON-TARGETplus CPSF4 siRNA	Dharmacon	012292-01
Random primers for reverse transcription	Invitrogen	48190011
qPCR primers	This paper and (Vilborg et al., 2015)	See Table S2
Software and Algorithms		
STAR	(Dobin et al., 2013)	https://github.com/alexdobin/STAR
mNET_snr	(Nojima et al., 2016)	https://github.com/tomasgomes/mNET_snr

(Continued on next page)

Continued

REAGENT or RESOURCE	SOURCE	IDENTIFIER
deepTools	(Ramírez et al., 2014)	https://github.com/deeptools/deepTools
Integrative Genomics Viewer (IGV)	(Robinson et al., 2011)	http://software.broadinstitute.org/software/igv/
R: A language and environment for statistical computing	R Foundation for Statistical Computing	https://www.R-project.org/
Other		
Genome sequence, primary assembly (GRCh38)	Genome Reference Consortium	ftp://ftp.sanger.ac.uk/pub/gencode/Gencode_human/release_27/GRCh38.primary_assembly.genome.fa.gz
GENCODE v27 human genome annotations	(Harrow et al., 2012)	https://www.gencodegenes.org/releases/27.html

CONTACT FOR REAGENT AND RESOURCE SHARING

Further information and requests for resources and reagents should be directed to and will be fulfilled by the Lead Contact, Prof. Ervin Fodor (ervin.fodor@path.ox.ac.uk)

EXPERIMENTAL MODEL AND SUBJECT DETAILS

Human adenocarcinoma alveolar basal epithelial (A549) cells were maintained in Dulbecco's Modified Eagle Medium (DMEM, Sigma-Aldrich) supplemented with 10% fetal calf serum (FCS). Madin-Darby Bovine Kidney (MDBK) and Madin-Darby Canine Kidney (MDCK) epithelial cells were maintained in Minimum Essential Medium (MEM, Sigma-Aldrich) supplemented with 2 mM L-glutamine and 10% FCS. Human embryonic kidney 293TRex/NS1 and 293TRex/NS1mut cell lines (Davidson et al., 2014) were maintained in DMEM supplemented with 10% FCS. Viral stocks of influenza A/WSN/33 (WSN) (H1N1) and A/PR/8/34 (H1N1) were produced by infecting MDBK cells at a multiplicity of infection (MOI) of 0.001 in DMEM. Stocks of A/Udorn/72 (H3N2) and B/Florida/04/2006 were produced by infecting MDCK cells at an MOI of 0.001. Trypsin was added to culture media at a concentration of 1 ug/ml for the production of A/PR/8/34, and 2 ug/ml for the production of B/Florida/04/2006 and A/Udorn/72. Stocks of mutant A/Udorn/72:NS1Δ99 virus were grown as described previously (Jackson et al., 2010).

METHOD DETAILS

Infection, Expression, and Osmotic Shock Experiments

For infection experiments, A549 cells were grown to 70%–80% confluency in 15 cm dishes, and infected at an MOI of 5 in DMEM. For all experiments, uninfected control cells were included which were mock-infected using DMEM. Cells were harvested at 12 hours post-infection for all viruses except WSN, which was harvested at 6 hours post-infection. Cells were harvested by washing with 20 mL of ice-cold PBS, collected by scraping, and transferred to 15 mL tubes. Cells were pelleted by centrifuging at 1000 x g at 4°C for 5 minutes, aspirating the supernatant, and flash-frozen in liquid nitrogen. Samples were stored at −80°C until use. Infections of 293TRex/NS1 cells with WSN were also carried out at an MOI of 5, with cells harvested at 4.5 hours post-infection by scraping first, then pelleting and resuspending twice in ice-cold PBS before flash-freezing in liquid nitrogen. For NS1 expression experiments, culture media was supplemented with 1 μg/ml tetracycline (Sigma) and cells were harvested after 24 hours. Osmotic shock experiments were carried out as described (Vilborg et al., 2015) by supplementing media with 80 mM potassium chloride and harvesting cells after 1 hour. Knockdown of CPSF30 was performed by transfecting 293TRex/NS1 cells at 25% confluency with SMARTpool ON-TARGETplus CPSF4 siRNA (L-012292-01, Dharmacon) at a final concentration of 30 nM using Lipofectamine RNAiMAX (Invitrogen), following the manufacturer's protocol. Control knockdowns (siLuc) were performed in parallel. Cells were harvested after 72 hours. Knockdown was confirmed by western blot for CPSF30 (A301-584A, Bethyl Laboratories) and actin as a control (A2006, Sigma). For the detection of DoG transcripts, total RNA was extracted from infected cells with TRIzol, and 125ng RNA was reverse transcribed with SuperScript III and 1.5ug random hexamers (Invitrogen) according to the manufacturer's protocol. qPCR was performed on a StepOnePlus instrument (ABI) using Brilliant III Ultra-Fast SYBR Green QPCR Master Mix (Agilent) and primer pairs for DoG transcripts (see Table S2 for sequences).

Sample Preparation for mNET-seq Experiments

Sample preparation for mNET-seq was performed as previously described (Nojima et al., 2016). Briefly, A549 cells were thawed on ice, lysed to pellet nuclei, and the nuclei were then lysed to precipitate chromatin. The chromatin pellet was digested with

micrococcal nuclease (NEB) for 4-5 minutes at 37°C and the supernatant containing the open chromatin fraction was incubated for 1 hour with magnetic bead-bound antibodies to either Pol II total CTD (MABI601, MBL) or Pol II Ser5P CTD (MABI603, MBL). After washing, Pol II-bound RNA was 3′-phosphorylated *in situ*, extracted using TRIzol (Invitrogen), and size-selected on a denaturing polyacrylamide gel to obtain fragments 35-100nt long. For mNET-seq, 293TRex/NS1 cells were prepared in the same manner, except micrococcal nuclease digestion was performed for 140 s, and RNA was isolated following 3′ phosphorylation using the Quick-RNA microprep kit (Zymo Research) and 17-200nt RNA was size-selected according to the manufacturer's protocol.

Library Preparation and Sequencing for mNET-seq Experiments

Libraries suitable for Illumina sequencing were prepared from RNA extracted following mNET-seq sample preparation using the TruSeq Small RNA Library Preparation kit (Illumina) for A549 cells, or the NEBNext Small RNA Library Preparation kit (NEB) for 293TRex/NS1 cells. Sequencing libraries were amplified by 15 cycles of PCR and size-selected on a 6% Novex TBE PAGE gel according to the manufacturers' instructions to remove primer-dimers. Paired-end sequencing (2x75bp) was performed on an Illumina HiSeq 2500 in Rapid Mode, or an Illumina HiSeq 4000 instrument at the Oxford Genomics Centre, Wellcome Trust Centre for Human Genetics (Oxford, UK).

Data Processing for mNET-seq

The overall pipeline for processing mNET-seq data was followed as previously described (Nojima et al., 2016), with some modifications. Briefly, sequencing reads were trimmed to remove any contaminating adaptor sequences using *cutadapt* 1.8.3 (Martin, 2011), and the resulting reads were mapped with to the GRCh38 genome using *STAR* (Dobin et al., 2013). The resulting BAM files were processed to extract the precise 3′ end position of each sequenced RNA fragment, and single-nucleotide mNET-seq profiles of Pol II occupancy were produced using the *bamCoverage* function of *deepTools* (Ramírez et al., 2014).

QUANTIFICATION AND STATISTICAL ANALYSIS

mNET-seq profiles were visualized with Integrative Genomics Viewer (IGV) (Robinson et al., 2011). Metagene analysis of Pol II occupancy was performed using transcription units as determined from the GENCODE v27 genome annotations (Harrow et al., 2012), using the *computeMatrix* and *plotProfile* functions of *deepTools*. The full list of all genes used for meta-profile analysis is given in Table S1; some genes were excluded from the meta-profiles on the basis that they are not expressed in the given cell line or do not have a single, dominant poly(A) site based on published data (Neve et al., 2016). The exact numbers of genes used for all meta-profiles and statistical analyses are given in each figure. Gene-wise Pol II occupancy of regions downstream of TSSs, and up and downstream of poly(A) sites was computed using the *computeMatrix* function of *deepTools*. A Pol II Depletion Index for each gene was calculated by taking the $\log_2$ ratio between Pol II occupancy in the interval TSS+1kb to TSS+2kb and the Pol II occupancy in the interval TSS+4kb to TSS+6kb. Gene body occupancy and a Pol II Pause Escape Index for each gene was calculated by as previously described (Schlackow et al., 2017). A Read-Thru Index for each gene was calculated by taking the $\log_2$ ratio between Pol II occupancy in the 2 kb downstream of the poly(A) site and the Pol II occupancy in the 2kb upstream of the poly(A) site. Genes with less than 10 reads in either case were excluded from analysis. A long-range Read-Thru Index was calculated by taking the $\log_2$ ratio between Pol II occupancy in the 2kb upstream of the poly(A) site and the Pol II occupancy in the interval poly(A) site+8kb to poly(A) site+10kb. The level of Pol II occupancy and the statistical indices above were compared between samples, and P values computed, using the Wilcoxon Rank Sum (Mann-Whitney) Test via the *wilcox.test* function in *R.* Published lists of DoG transcript genes during osmotic shock (Vilborg et al., 2017) were used for comparisons to influenza virus infection, in addition to the osmotic shock mNET-seq data presented here. Scatterplots were generated using the *ggplot2* and *hexbin* packages in *R*, and the and gene-wise Pearson's correlation between samples was calculated using the *cor.test* function in *R*.

DATA AND SOFTWARE AVAILABILITY

The accession number for the sequencing data reported in this paper is NCBI SRA: SRP132032.

Cell Reports, Volume 23

Supplemental Information

Influenza Virus Mounts a Two-Pronged Attack

on Host RNA Polymerase II Transcription

David L.V. Bauer, Michael Tellier, Mónica Martínez-Alonso, Takayuki Nojima, Nick J. Proudfoot, Shona Murphy, and Ervin Fodor

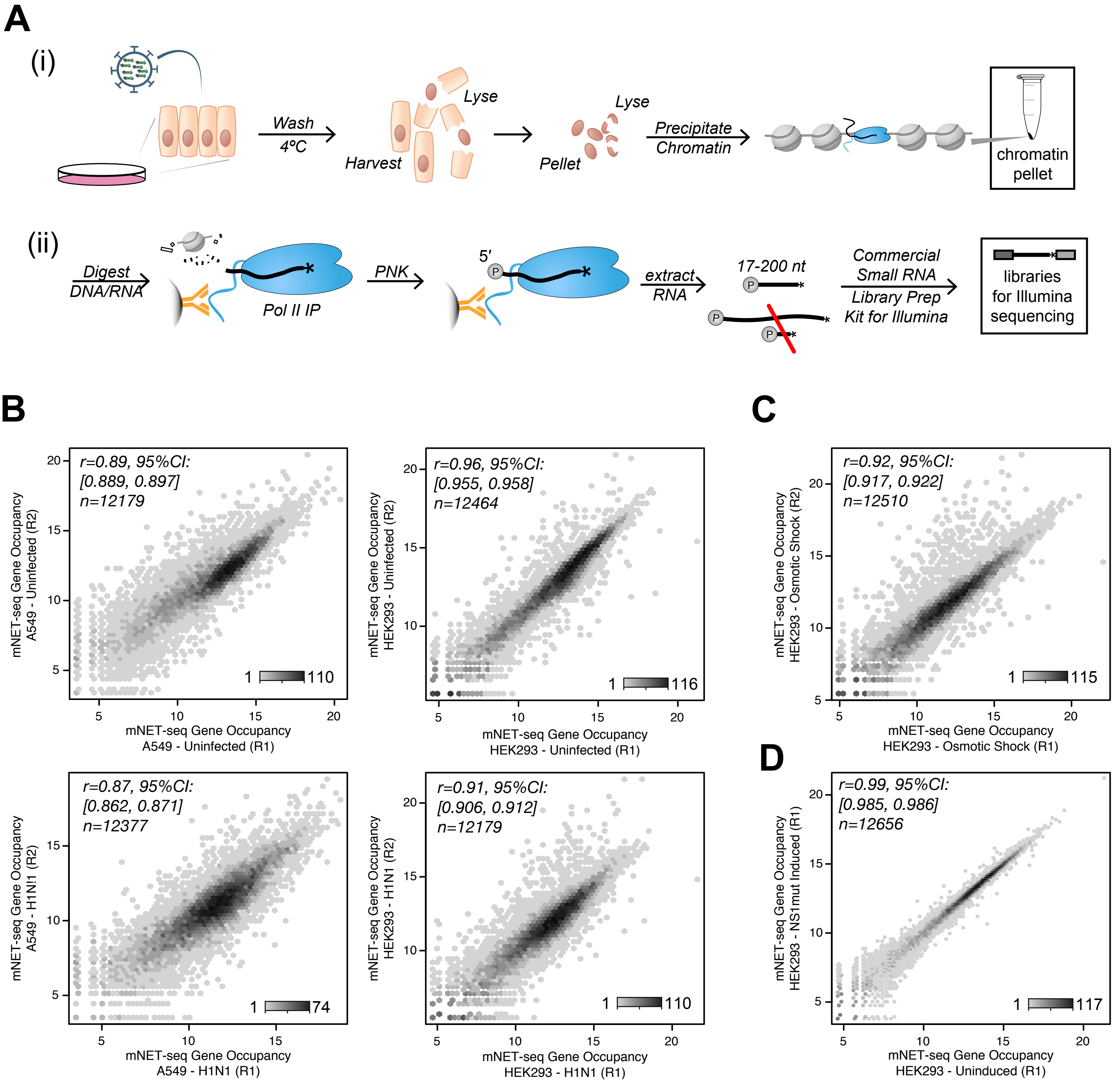

Figure S1. Mammalian Nascent Elongating Transcript - Sequencing (mNET-seq) Generates Highly Reproducible Profiles of Pol II Occupancy Across the Genome. Related to Figure 1

(A) Outline of mNET-seq (i) sample processing and (ii) library preparation, based on the method described in (Nojima et al., 2015) and the protocol described in detail in (Nojima et al., 2016). See STAR Methods for further details.

(B) Comparison of mNET-seq results (gene body Pol II occupancy) between biological replicates (R1 and R2) in A549 (left) and HEK293 (right) cells either uninfected (top) or H1N1-infected (bottom).

(C) Comparison of mNET-seq results (gene body Pol II occupancy) between biological replicates of osmotic shock treatment in HEK293 cells.

(D) Comparison of mNET-seq results (gene body Pol II occupancy) between uninduced HEK293 cells and cells in which expression of a CPSF30 binding mutant of the influenza virus NS1 protein (NS1mut) was induced.

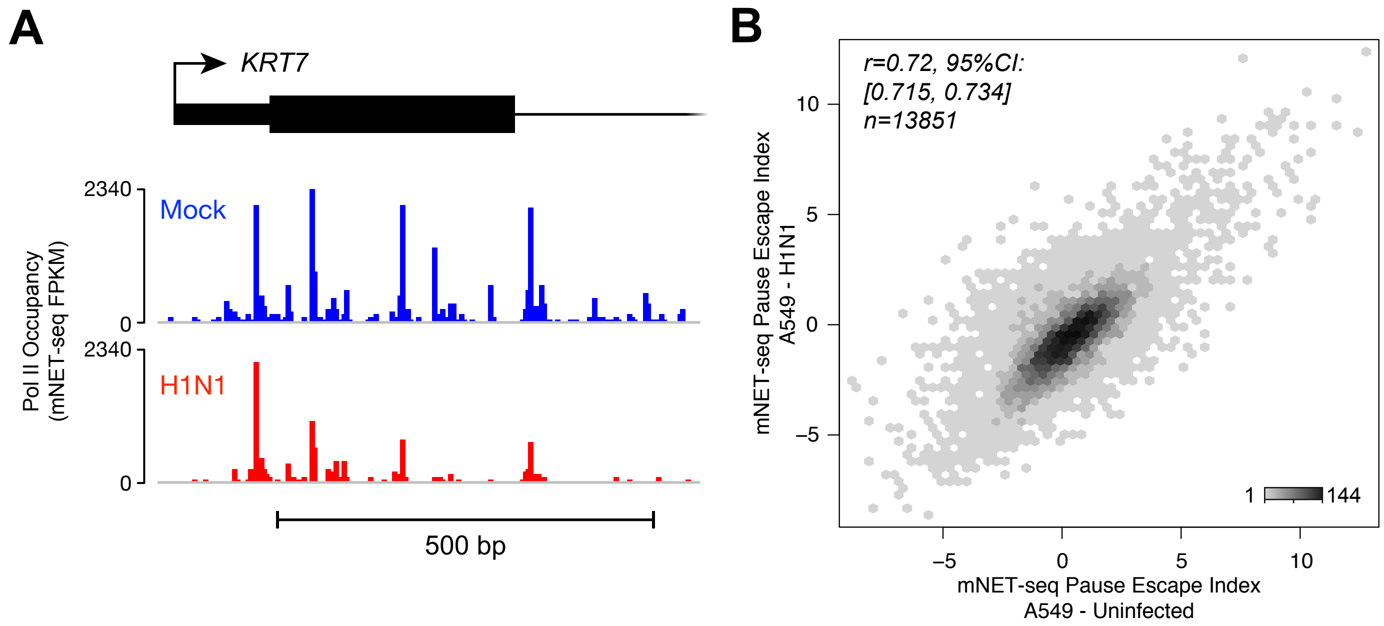

Figure S2. Pol II Occupancy Tails Off Downstream of Transcription Start Sites During Influenza Infection. Related to Figure 2.

(A) mNET-seq profiles of Pol II occupancy at the start of the protein-coding gene *KRT7*. Influenza virus infection (H1N1) causes a 'tailing-off' of Pol II occupancy in the body of the gene.

(B) The Pause Escape Index (ratio between the amount of promoter-proximally paused Pol II in the TSS region and amount of Pol II in the gene body, see STAR Methods) was calculated for each gene, and compared between uninfected and H1N1-infected samples. The linear correlation (Pearson's r=0.72, 95% confidence interval = [0.715, 0.734]) reflects the 'tailing-off' of Pol II and also suggests that FluPol does not preferentially target genes with different amounts of promoter-proximally-paused Pol II, which would generate a non-linear correlation.

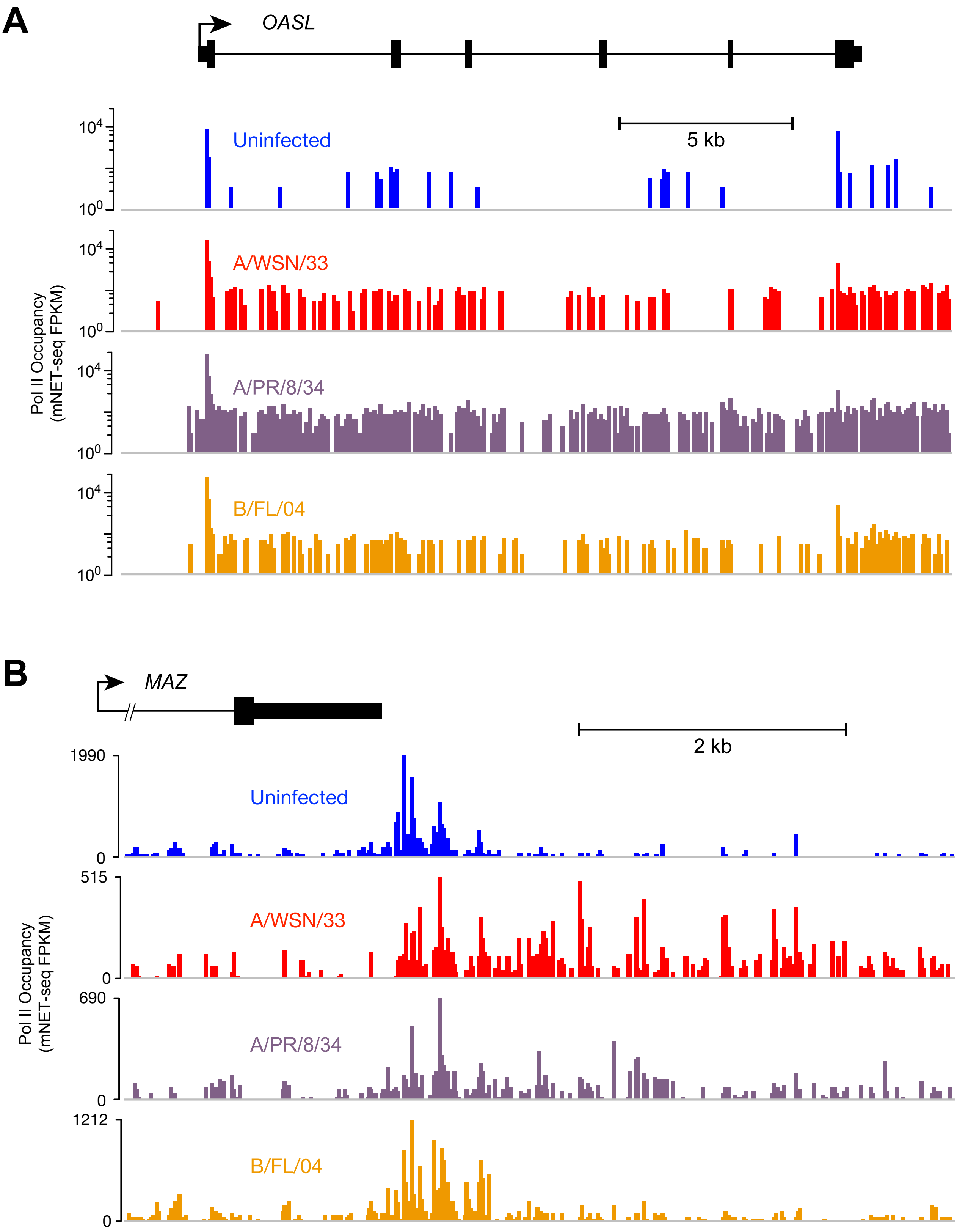

Figure S3. Transcriptional Response to Influenza Virus Infection. Related to Figure 5.
(A) mNET-seq profiles of Pol II occupancy along the antiviral gene *OASL* , in uninfected and infected (A/WSN/33, A/PR/8/34, and B/Florida/04) cells. Pol II occupancy along *OASL* is increased during viral infection, suggesting that cells respond to viral infection. Note that the y-axis is presented on a logarithmic scale.
(B) mNET-seq profiles of Pol II occupancy along the protein-coding gene *MAZ* show the same pattern of termination defect as on *KRT7* (Figure 5). The unexpressed downstream *PRRT2* gene is not shown in the annotation.

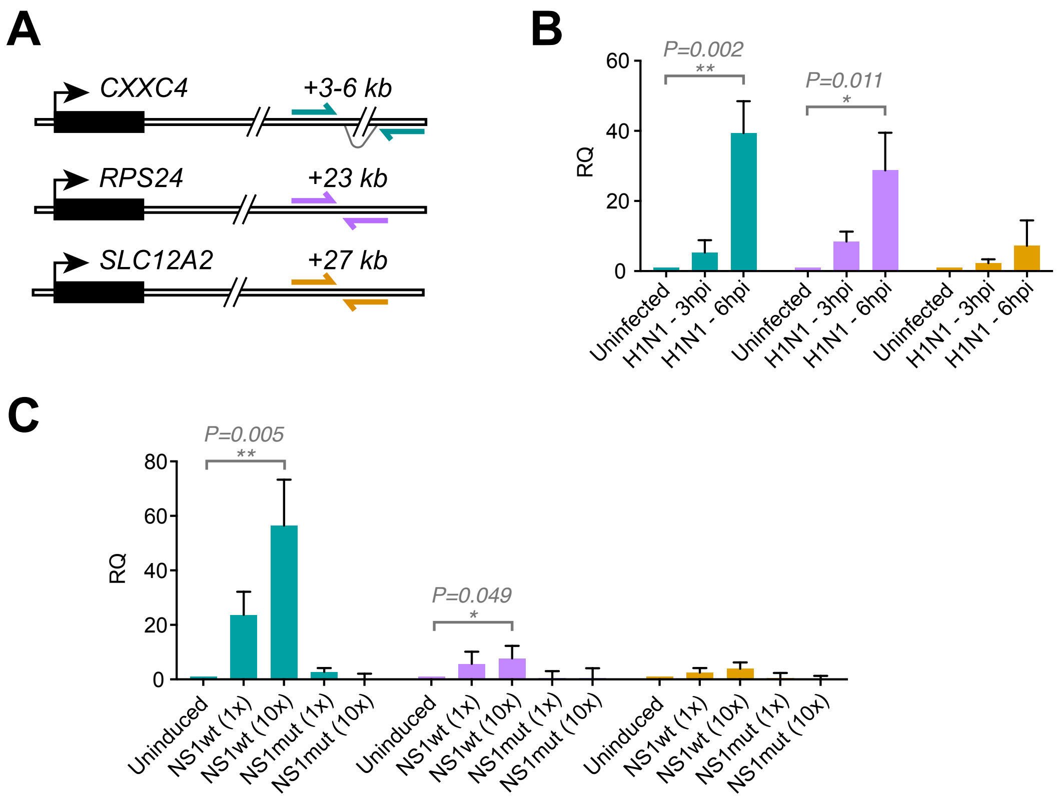

Figure S4. Downstream-of-Gene transcripts (DoGs) Are Produced During Influenza Virus Infection and Viral NS1 Protein Expression. Related to Figure 6.
(A) RT-qPCR scheme for the detection of DoGs (see STAR Methods) downstream of *CXXC4*, *RPS24*, and *SLC12A2*. The grey connecting arc downstream of *CXXC4* represents a reported splice junction (Vilborg et al., 2015).
(B) Quantitation of DoGs present during influenza virus infection in A549 cells at 3 and 6 hours post infection (hpi). Error bars represent standard deviation from three biological replicates.
(C) Quantitation of DoGs present following induction of NS1wt or NS1mut protein expression in HEK293 cells. (1x) indicates 1 µg/ml tetracycline, (10x) indicates 10 µg/ml tetracycline used to induce protein expression. Error bars represent standard deviation from three biological replicates.

Name	Sequence (5′ to 3′)	Reference
doCXXC4F	CAGTGAAGCTCTTCGATTTCAA	(Vilborg et al., 2015)
doCXXC4R	CCCCCTATATACCGAAGTGGA	(Vilborg et al., 2015)
SLC12A2_tdF	GAAACTGCTGTTGGAAGCGG	
SLC12A2_tdR	TGGCCTTGGATTAGCATCCG	
RPS24_td23_F	CTTGGATGCCATGTTGCCAG	
RPS24_td23_R	CAGACCAAAAGCGGAGCCTA	
18s_F	ACCCGTTGAACCCCATTCGTGA	
18s_R	GCCTCACTAAACCATCCAATCGG	

Table S2. Primer oligonucleotide sequences used for RT-qPCR detection of DoG transcripts. Related to STAR Methods.
